# A Phase 1/2 Dose-Ranging Safety and Immunogenicity Study of mRNA-Based Candidate Pandemic Influenza Vaccines in Healthy Adults

**DOI:** 10.1093/cid/ciag278

**Published:** 2026-04-25

**Authors:** Catherine A Cosgrove, Andrei Avanesov, Dondi Rust, Seun Osibajo, Kingston Kang, Brett Leav, the ODYSSEY Study Group, Paul Heath, Saul Faust, Marta Boffito, Christopher Duncan, Fiona Burns, Amardeep Heer, Philip Kalra, Rajeka Lazarus, Adrian Palfreeman, Tommy Rampling, Anil Shenoy, Anna Goodman, Kamran Hussain, Orod Osanlou, Christopher Green, Jeremy Turner, Andrew Freedman, Rebecca Sutherland, David Fried, Rebecca Rivard, Nelia Sanchez-Crespo, Carlos Fierro, Paul Bradley, Lisa Holloway, Earl F Martin, Katie Ann Julien, Jay Meyer

**Affiliations:** Institute of Infection and Immunity & Vaccine Institute, City St George’s, University of London, United Kingdom & St George’s University Hospital NHS Trust, London, United Kingdom; Clinical Operations and Clinical Biomarkers, Moderna, Inc., Cambridge, Massachusetts, USA; Clinical Operations and Clinical Biomarkers, Moderna, Inc., Cambridge, Massachusetts, USA; Clinical Operations and Clinical Biomarkers, Moderna, Inc., Cambridge, Massachusetts, USA; Clinical Operations and Clinical Biomarkers, Moderna, Inc., Cambridge, Massachusetts, USA; Clinical Operations and Clinical Biomarkers, Moderna, Inc., Cambridge, Massachusetts, USA

**Keywords:** mRNA, pandemic influenza vaccine, phase 1/2 clinical trial, safety, immune response

## Abstract

**Background:**

Influenza A viruses pose a persistent pandemic threat. We report safety, reactogenicity, and immunogenicity findings for mRNA-1018 pandemic influenza vaccine candidates from a phase 1/2 study in healthy adults.

**Methods:**

In part A, participants were randomized to receive 1 of 4 mRNA-1018 candidates at 1 of 3 dose levels across 2 influenza A groups: (1) H5N8/H5-only or (2) H7N9/H7-only. Part B participants were randomized to receive H5-only-CG. The primary objectives were to evaluate the safety and reactogenicity. The secondary objectives included evaluation of humoral immunogenicity through day 205 by hemagglutination inhibition (HAI), neuraminidase inhibition, and microneutralization assays.

**Results:**

Parts A and B comprised 1195 and 304 dosed participants, respectively. Overall, solicited local adverse reactions (ARs) within 7 days of vaccination occurred in 76.8% of participants across vaccine candidates and dose levels, most commonly injection-site pain. Solicited systemic ARs were reported in 62.8% of participants, most frequently fatigue and headache. Postvaccination immune responses, assessed absolutely, by HAI titers and dynamically, by seroconversion rates, tended to increase with vaccine dose. H5-based candidates induced stronger strain-specific HAI.

**Conclusions:**

Vaccine candidates were sufficiently well tolerated and immunogenic. Further development of mRNA pandemic influenza vaccines is warranted.

Clinical Trials Registration. NCT05972174

## Teaser

This study demonstrated that 5 mRNA-1018 vaccine candidates targeting 2 influenza A virus groups elicited strong, sustained immune responses in healthy adults, with no safety concerns identified, supporting continued development of mRNA pandemic influenza vaccines.

Influenza A viruses pose a persistent pandemic threat due to their zoonotic potential and antigenic shift capability, enabling emergence of novel strains to which the global population has little or no immunity [1, 2]. Absence of population-level immunity facilitates rapid transmission of pandemic influenza A, contributing to a higher cumulative incidence of severe disease and mortality, with greater societal disruption, compared with seasonal influenza [2, 3]. High-risk populations for severe outcomes from pandemic influenza A include infants, the elderly, individuals with comorbidities, and potentially healthy young adults, as evidenced by past pandemics [3].

In the past decade, high-pathogenicity avian influenza (HPAI) viruses have repeatedly crossed the species barrier from poultry to infect humans [2, 4]. The A(H7N9) virus, first detected in 2013, caused >1500 human infections and >600 deaths across multiple epidemic waves [2]. HPAI H5 viruses, particularly A(H5N1), have caused global outbreaks among poultry and wild birds, with sporadic cases reported in mammals, including humans [2, 4]. Most recently, transmission to humans from an ongoing outbreak among dairy cattle has been reported [5]. Between February 2024 and September 2025, 70 A(H5N1) human infections have been reported in the United States, but no human-to-human transmission has been identified [5, 6]. These events underscore the ongoing pandemic potential of avian influenza A viruses and highlight the importance of pandemic preparedness [7].

Vaccination may be the most effective method of mitigating disease severity and reducing future influenza A pandemic risk [7, 8]. Several pandemic influenza vaccines are licensed and authorized for use in the United States and Europe in the event of a pandemic and are manufactured using either egg- or cell-based platforms [9–14]. The mRNA vaccine platform provides advantages over conventional vaccine technologies, offering a promising strategy for pandemic preparedness through rapid design, scalable production, and precise antigenic matching, enabling timely and effective responses to emerging threats [15–17]. In a recently completed phase 3 study in adults ≥ 50 years, mRNA-1010, an investigational seasonal influenza vaccine, demonstrated superior relative vaccine efficacy compared with a licensed standard-dose comparator (NCT06602024) [18]. The demonstration of superior clinical benefit, which correlated with superior immune responses, and an acceptable safety and tolerability profile similar to a prior phase 3 study of mRNA-1010 (NCT05827978) [16], supports the development of pandemic influenza vaccines manufactured using this platform.

In the present study in adults ≥ 18 years, we present the final analysis of safety, reactogenicity, and immunogenicity through 7 months after vaccination with 1 of 5 mRNA-1018 vaccine candidates targeting 2 different influenza A virus groups with pandemic potential (group 1, H5N8 or H5N1; group 2, H7N9) [19].

## METHODS

### Study Design and Participants

This phase 1/2, randomized, observer-blind, parallel, dose-ranging study evaluated the safety, reactogenicity, and immunogenicity of 5 mRNA-1018 candidates in adults ≥ 18 years at 9 sites in the United States and 18 in the United Kingdom (NCT05972174 [20]). Participants were stratified by age (18 to <65 years and ≥65 years). The study comprised 2 parts that enrolled and ran concurrently. Part A evaluated 4 mRNA-1018 candidates (H5N8, H7N9, H5-only, and H7-only); part B evaluated a single mRNA-1018 candidate (H5-only-CG).

Eligible participants were healthy adults ≥ 18 years. A full list of inclusion/exclusion criteria is contained in the Supplementary Data. In part A, participants were evenly randomized to receive 1 of 4 mRNA-1018 candidates at 1 of 3 dose levels across 2 influenza A virus groups: H5N8 or H5-only (group 1) or H7N9 or H7-only (group 2). H5N8 and H7N9 candidates were administered at doses of 25, 50, or 100 µg; H5-only and H7-only were administered at 12.5, 25, or 50 µg. In part B, 304 participants were evenly randomized to receive 12.5, 25, or 50 µg of H5-only-CG (group 1; Supplementary Figure 1). Randomization and blinding are detailed in the Supplementary Data. All mRNA-1018 candidates were administered intramuscularly as a 2-dose primary series, 21 days apart (days 1 and 22); the final study visit occurred 6 months after dose 2 (day 205, month 7).

The study was conducted in accordance with the protocol, applicable laws, and regulatory requirements, as well as the International Council for Harmonisation Good Clinical Practice guidelines and the consensus ethical principles derived from international guidelines, including the Declaration of Helsinki and Council for International Organizations of Medical Sciences International Ethical Guidelines. The protocol was approved by the institutional review boards/independent ethics committees (Advarra, Columbia, MD, USA; approval number: Pro00071555) prior to study initiation, and written informed consent was obtained from all participants before enrollment.

### Vaccines

The nucleic acid sequences of the surface glycoproteins hemagglutinin (HA) and neuraminidase (NA) used in this study were derived from the following vaccine candidate viruses: part A, H5N8 (A/Astrakhan/3212/2020) and H7N9 (A/Anhui/1/2013), and part B, H5N1 (A/chicken/Ghana/AVL-763_21VIR7050-39/2021). The mRNA-1018 candidates H5-only, H7-only, and H5-only-CG included mRNAs encoding only HA. These candidates differ in the HA sequences that they encode, namely, H5N8 (A/Astrakhan/3212/2020) and H5N1 (A/chicken/Ghana/AVL-763_21VIR7050-39/2021), respectively. Additional details can be found in the Supplementary Appendix. The HA- and NA-containing candidates contain the 2 sequences in a 1:1 mass ratio. The vaccines comprised mRNAs formulated in lipid nanoparticles consistent with the standard manufacturing platform used for the Sponsor's licensed vaccines [21–23].

### Study Objectives

The primary objectives were to evaluate the safety and reactogenicity of each mRNA-1018 candidate at different dose levels. The secondary objectives were to evaluate humoral immunogenicity by hemagglutination inhibition (HAI) and NA inhibition (NAI) assays against vaccine-matched influenza A strains after 2 doses (day 43 vs day 1 [baseline]), after 1 dose (day 22 vs day 1), and after the second dose (day 29 vs day 1). Exploratory objectives included evaluation of humoral immunogenicity against vaccine-matched influenza A strains after the first dose (day 8 vs day 1), persistence of humoral immunogenicity (day 205 vs day 1), and neutralizing activity by single-cycle microneutralization (MN) assay.

### Reactogenicity and Safety Assessments

Endpoints included the incidence and severity of solicited local and systemic adverse reactions (ARs) assessed via an electronic diary for 7 days after each injection; unsolicited adverse events (AEs) for 21 days after each injection; and AEs leading to discontinuation, serious AEs (SAEs), AEs of special interest (AESIs), and medically attended AEs (MAAEs) throughout the study (to day 205 [end of study]). The grading scale used to assess reactogenicity and safety is detailed in Supplementary Table 1.

### Immunogenicity Assessments

Blood samples for immunogenicity assessments were collected on days 1, 8, 22, 29, 43, and 205. Humoral immunogenicity endpoints were measured by qualified HAI, NAI, and MN assays (detailed in the Supplementary Data).

### Statistical Analyses

No formal statistical hypotheses were tested. The sample size (detailed in the Supplementary Data) was considered sufficient to provide a descriptive summary of the safety, reactogenicity, and immunogenicity of different dose levels of the studied mRNA-1018 candidates. The safety set included all participants who received vaccine. The per-protocol set was the primary analysis population used to assess immunogenicity (defined in the Supplementary Data). Descriptive statistics were used to summarize safety and immunogenicity data; additional details can be found in the Supplementary Data. Antibody titers below the assay lower limit of quantification (LLOQ) were replaced by 0.5 × LLOQ.

## RESULTS

### Participants

A total of 2102 healthy adults ≥ 18 years were screened, and 1506 (71.7%) were randomized between 10 July 2023 and 16 July 2024 (part A, N = 1202; part B, N = 304). First and second injections were received by 99.4% and 95.4% of participants, respectively, in part A and by 100% and 96.1%, respectively, in part B (Figure 1). Overall, 1127 (93.8%) participants completed part A and 274 (90.1%) completed part B. The main reasons for participant discontinuation in both study parts were loss to follow-up and withdrawal of consent. Across vaccine groups (parts A and B), the median age range was 52.0–56.0 years, and most participants were female (57.1% and 58.6%, respectively), non-Hispanic (92.3% and 70.4%, respectively), and White (79.6% and 76.6%, respectively). Baseline characteristics were comparable between vaccine groups (Table 1). The median study duration range among participants was 201–203 days (first injection) and 179–180 days (second injection) across vaccine candidates in part A and 198 and 176 days, respectively, in part B.

**Figure 1.**
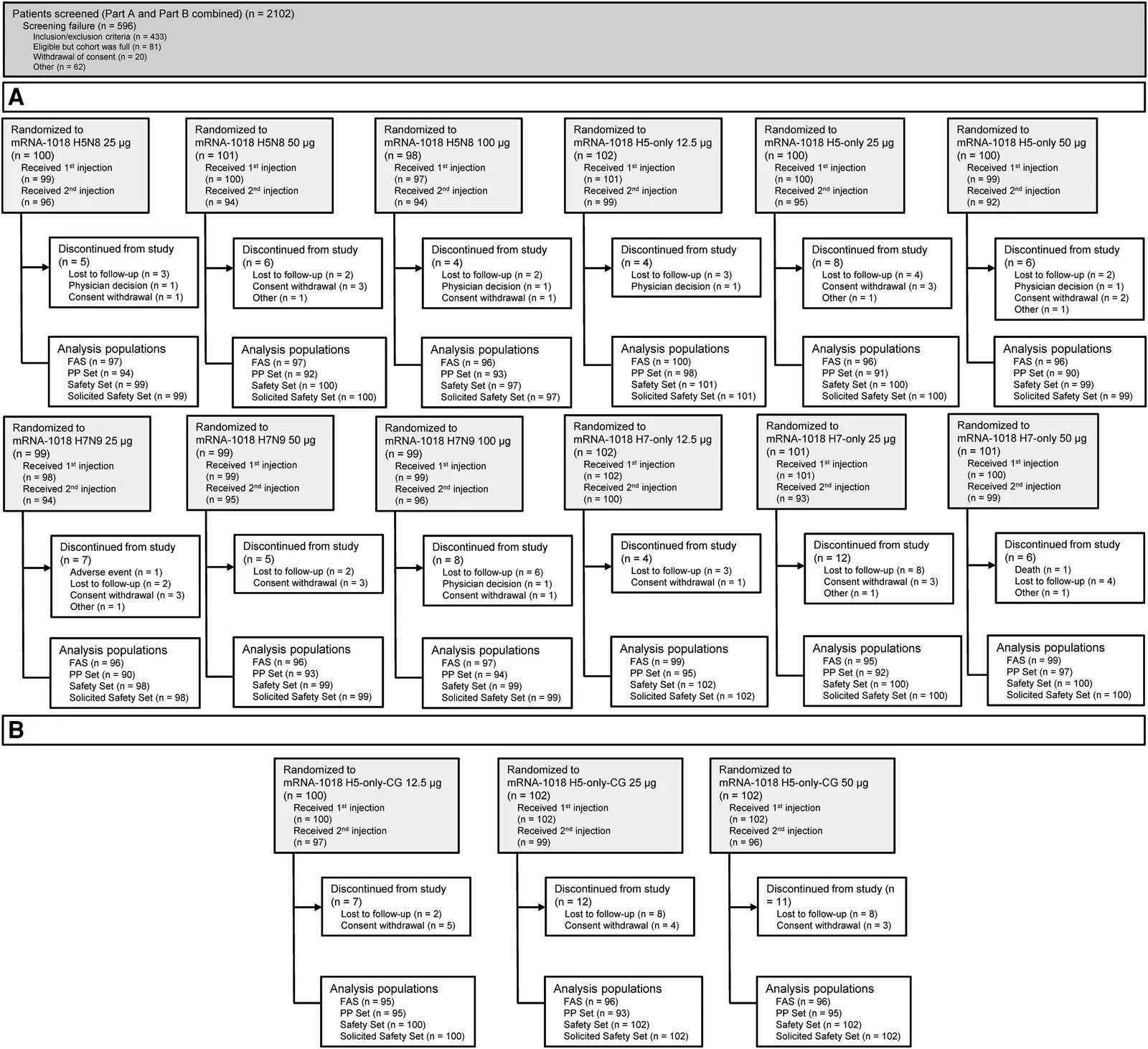
Participant disposition in (*A*) part A (H5N8, H5-only, H7N9, and H7-only mRNA-1018) and (*B*) part B (H5-only-CG mRNA-1018). The FAS consisted of all randomized participants who received ≥1 dose of the study vaccine, had baseline (day 1) data available for those analyses that required baseline data, and had ≥1 postinjection assessment for the primary analysis endpoint. The PP set consisted of all FAS participants who complied with the injection schedule, complied with the timings of humoral immunogenicity blood sampling and had a baseline and ≥1 evaluable postinjection assessment corresponding to the immunogenicity analysis objective (immunogenicity endpoints), did not have influenza infection at baseline through day 43 (as confirmed by the study investigational site), and had no major protocol deviations impacting immune response during the period corresponding to the humoral immunogenicity analysis objective (immunogenicity endpoints). The safety set consisted of all randomly assigned participants who received the investigational product. The solicited safety set consisted of all participants in the safety set who contributed any solicited adverse reaction data (ie, had ≥1 postbaseline solicited safety [eDiary] assessment). Abbreviations: FAS, full analysis set; PP, per-protocol.

**Table 1.** Participant Demographics and Baseline Characteristics by mRNA-1018 Vaccine Candidate Group (All Dose Levels Combined), Safety Set

	mRNA-1018 Vaccine Candidate Groups				
	Part A				Part B
	H5N8 (N = 296)	H5-only (N = 300)	H7N9 (N = 296)	H7-only (N = 303)	H5-only-CG (N = 304)
Age, years					
Mean (SD)	52.6 (16.4)	51.9 (17.1)	53.2 (16.5)	51.7 (17.6)	52.8 (16.5)
Median	54.0	52.0	56.0	55.0	54.5
Min, max	18, 90	18, 89	19, 89	19, 85	18, 82
18–64 y, n	204	208	204	208	207
≥65 y, n	92	92	92	95	97
Sex, n (%)					
Male	113 (38.2)	144 (48.0)	130 (43.9)	126 (41.6)	126 (41.4)
Female	183 (61.8)	156 (52.0)	166 (56.1)	177 (58.4)	178 (58.6)
Race, n (%)					
American Indian or Alaska Native	1 (0.3)	1 (0.3)	0	1 (0.3)	0
Asian	5 (1.7)	7 (2.3)	15 (5.1)	13 (4.3)	10 (3.3)
Black/African American	40 (13.5)	37 (12.3)	37 (12.5)	52 (17.2)	53 (17.4)
Native Hawaiian or Other Pacific Islander	1 (0.3)	0	1 (0.3)	0	1 (0.3)
Other	5 (1.7)	3 (1.0)	3 (1.0)	7 (2.3)	3 (1.0)
White	240 (81.1)	250 (83.3)	232 (78.4)	229 (75.6)	233 (76.6)
Not reported/unknown	1 (0.3)	1 (0.3)	2 (0.7)	1 (0.3)	2 (0.7)
Multiple	3 (1.0)	1 (0.3)	6 (2.0)	0	2 (0.7)
Ethnicity, n (%)					
Hispanic/Latino	17 (5.7)	23 (7.7)	18 (6.1)	21 (6.9)	88 (28.9)
Not Hispanic/Latino	276 (93.2)	273 (91.0)	274 (92.6)	280 (92.4)	214 (70.4)
Not reported/unknown	3 (1.0)	4 (1.3)	4 (1.4)	2 (0.7)	2 (0.7)
BMI, kg/m^2^					
Mean (SD)	27.8 (4.8)	28.2 (4.6)	27.9 (4.7)	28.2 (5.1)	28.6 (4.8)
Median	27.6	28.0	27.5	27.8	28.7
Min, max	18.2, 39.1	18.1, 38.7	18.8, 39.0	18.2, 39.0	18.1, 38.7

Abbreviations: BMI, body mass index; SD, standard deviation.

### Reactogenicity and Safety

Across the range of dose levels, solicited local ARs occurring within 7 days after either injection were reported by 78.5% (938/1195) of participants in part A (H5N8, 74.7%–92.8%; H5-only, 67.3%–91.9%; H7N9, 77.6%–89.9%; H7-only, 61.8%–80.0%) (Figure 2*A*) and 70.1% (213/304) of participants in part B (H5-only-CG, 67.6%–72.5%) (Figure 2*B*). The percentage of participants reporting any solicited local AR within 7 days after either vaccination dose generally increased with dose level within each vaccine group in parts A and B, with the highest incidence reported for H5N8 100 µg (92.8% [90/97]). The most frequently reported solicited local AR in each vaccine group across study parts was injection-site pain, followed by axillary swelling/tenderness (Supplementary Figure 2). Most solicited local ARs were grade 1–2 in severity. Incidence of grade 3 solicited local ARs in parts A and B was low (1.4% [17/1195] and 0.7% [2/304], respectively) and generally occurred in the higher-dose-level groups across vaccine candidates (Figure 2). Grade 3 local ARs included pain, erythema, and local and axillary swelling (Supplementary Figure 2). No grade 4 local ARs were reported. The median duration of solicited local ARs after the first and second injections was 2.0 days across vaccine groups.

**Figure 2.**
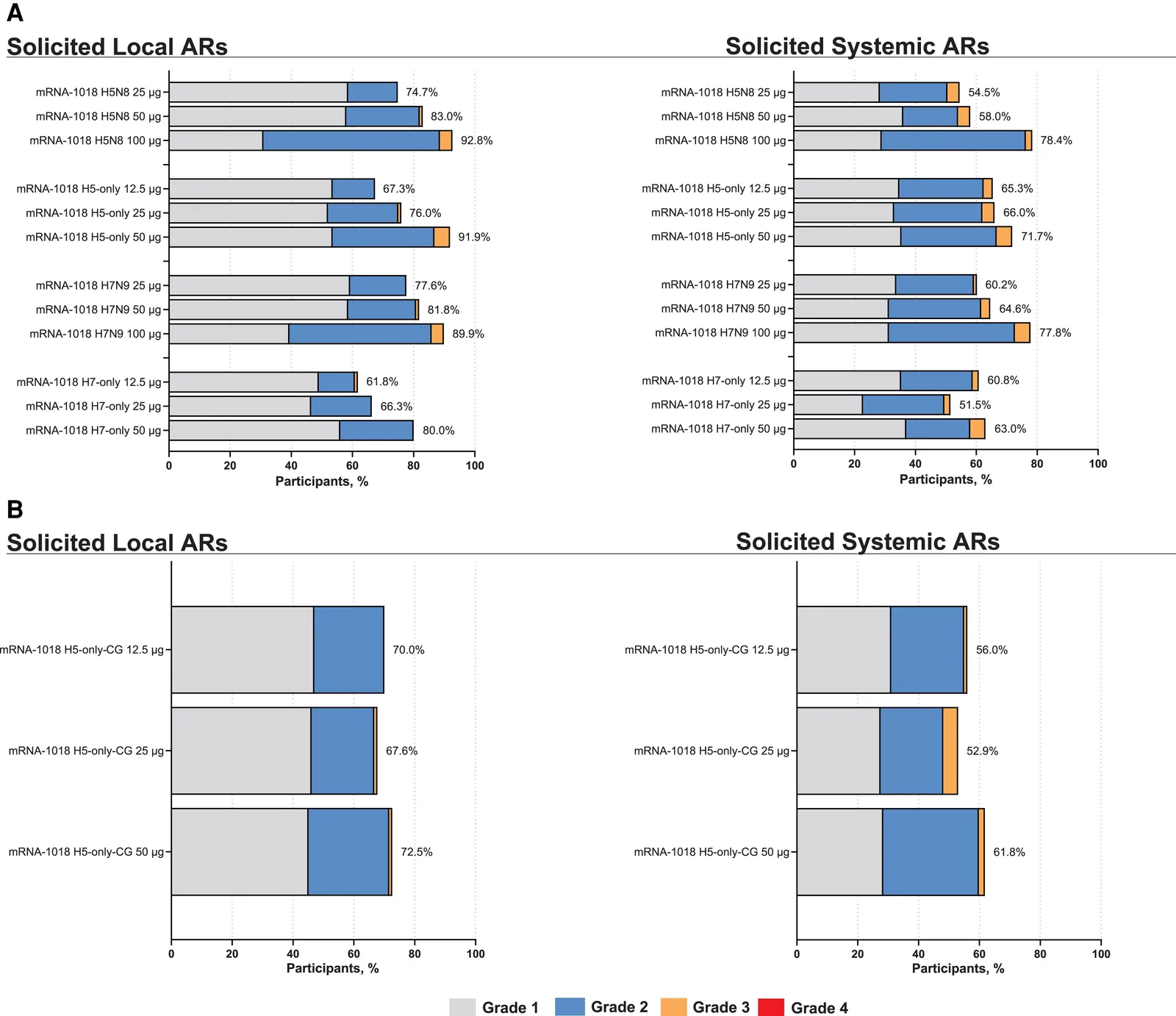
Percentages of participants reporting solicited local and systemic adverse reactions within 7 d after either the first or second mRNA-1018 injection in each vaccine candidate arm in the overall population in (*A*) part A and (*B*) part B, solicited safety set. The solicited safety set included all randomized participants who received the study injection and contributed any solicited adverse reaction data.

Solicited systemic ARs after any injection were reported by 64.3% (768/1195) of participants in part A (H5N8, 54.5%–78.4%; H5-only, 65.3%–71.7%; H7N9, 60.2%–77.8%, H7-only, 51.5%–63.0%) (Figure 2*A*) and 56.9% (173/304) in part B (H5-only-CG, 52.9%–61.8%) (Figure 2*B*). The percentage of participants reporting any solicited systemic ARs increased with dose level and was the highest in the H5N8 100 µg group in part A (78.4% [76/97]) and the H5-only-CG 50 µg group in part B (61.8% [63/102]). The most common solicited systemic ARs after either injection across vaccine candidates were fatigue and headache (Supplementary Figure 2). Most solicited systemic ARs were grade 1–2 in severity (Figure 2). Grade 3 events were infrequent (part A, 3.3% [40/1195)]; part B, 2.6% [8/304]) and were reported across all vaccine candidates as headache, fatigue, myalgia, chills, and arthralgia (Supplementary Figure 2). No grade 4 solicited systemic ARs were reported. The median duration of solicited systemic ARs after the first and second injections was 2.0 days across all vaccine groups (range, 1.0–3.0).

In part A, the incidence of solicited local and systemic ARs across all doses and vaccine groups was higher in those 18–64 years (local, 67.1%–97.0%; systemic, 55.7%–83.6%) compared with those ≥65 years (local, 50.0%–90.0%; systemic, 33.3%–74.2%) (Supplementary Figure 3*A*). In part B, incidence of ARs across all doses and vaccine groups was similar across age groups (≥65 years; local, 69.1%–74.3%; systemic, 52.2%–62.9% vs 18–64 years; local, 63.6%–71.9%; systemic, 50.0%–59.4%) (Supplementary Figure 3*B*).

The proportion of participants who experienced any unsolicited AE throughout the study after any vaccine dose across vaccine groups in parts A and B ranged from 31.9%–50.0%. Unsolicited AEs considered related to study vaccine during the overall observation period occurred in 2.3%–5.6% of participants in parts A and B (Supplementary Table 2). There was no apparent dose-related pattern within any group. Throughout the study, unsolicited AEs led to study discontinuation in 10 participants (0.8%) in part A and none in part B. One event of acute thyroiditis resulting in study vaccine discontinuation was considered vaccine related by the investigator (H5-only 50 µg group). Serious AEs were reported for 24 participants (2.0%) in part A and 4 (1.3%) in part B, one of which was considered vaccine related by the investigator (syncope; H5-only-CG 12.5 µg group). Twelve AESIs were reported, 2 of which were considered vaccine related by the investigator (2 cases of nonserious mild thrombocytopenia; H5-only 12.5 µg group [n = 1] and H5-only-CG 25 µg group [n = 1]). One death (due to toxic shock syndrome), assessed as not related to the study vaccine, was reported on day 139 for a 74-year-old male who had received two 50 µg doses of H7-only. No AESIs of myocarditis, pericarditis, or myopericarditis were reported in either study part. The overall percentage of participants reporting unsolicited AEs was similar across the 18–64 years and ≥65 years age groups in part A (47.8% vs 46.1%) and was lower in the younger age group in part B (27.1% vs 42.3%).

A summary of unsolicited AEs through 21 days after the first or second injections by vaccine group (all dose levels combined) is provided in Supplementary Table 3.

### Immunogenicity

On day 1, few participants in any vaccine group had detectable immune responses as assessed by HAI geometric mean titers (GMTs) against any of the pandemic influenza strains tested (Table 2 and Figure 3). In part A, day 43 HAI GMTs (95% CI; overall population) were 118.4 (95.3–147.0), 157.0 (128.9–191.2), and 249.1 (200.8–309.1) in the H5N8 25, 50, and 100 μg dose groups, respectively; 160.6 (131.9–195.6), 285.8 (238.0–343.3), and 239.4 (194.8–294.3) for the H5-only 12.5, 25, and 50 µg dose groups, respectively; 38.1 (31.5–46.1), 49.6 (40.9–60.2), and 87.7 (71.2–108.1) for the H7N9 25, 50, and 100 μg dose groups, respectively; and 23.6 (18.8–29.5), 37.5 (30.4–46.3), and 44.2 (35.1–55.7) for the H7-only 12.5, 25, and 50 µg dose groups, respectively. The percentage of participants meeting the criteria for seroconversion (defined as HAI titer ≥ 1:40 if baseline < 1:10 or 4-fold rise if baseline > 1:10, postvaccination) at day 43 in part A ranged from 91.3% to 98.9% for the H5-containing vaccine candidates and from 40.9% to 89.4% for the H7-containing candidates. In part B, day 43 HAI GMTs (95% CI) for the H5-only-CG vaccine were 203.9 (164.7–252.4), 236.7 (194.7–287.8), and 340.9 (283.1–410.6) in the 12.5, 25, and 50 μg dose groups, respectively. The percentage of participants meeting the criteria for seroconversion at day 43 ranged from 94.6% to 100.0%.

**Figure 3.**
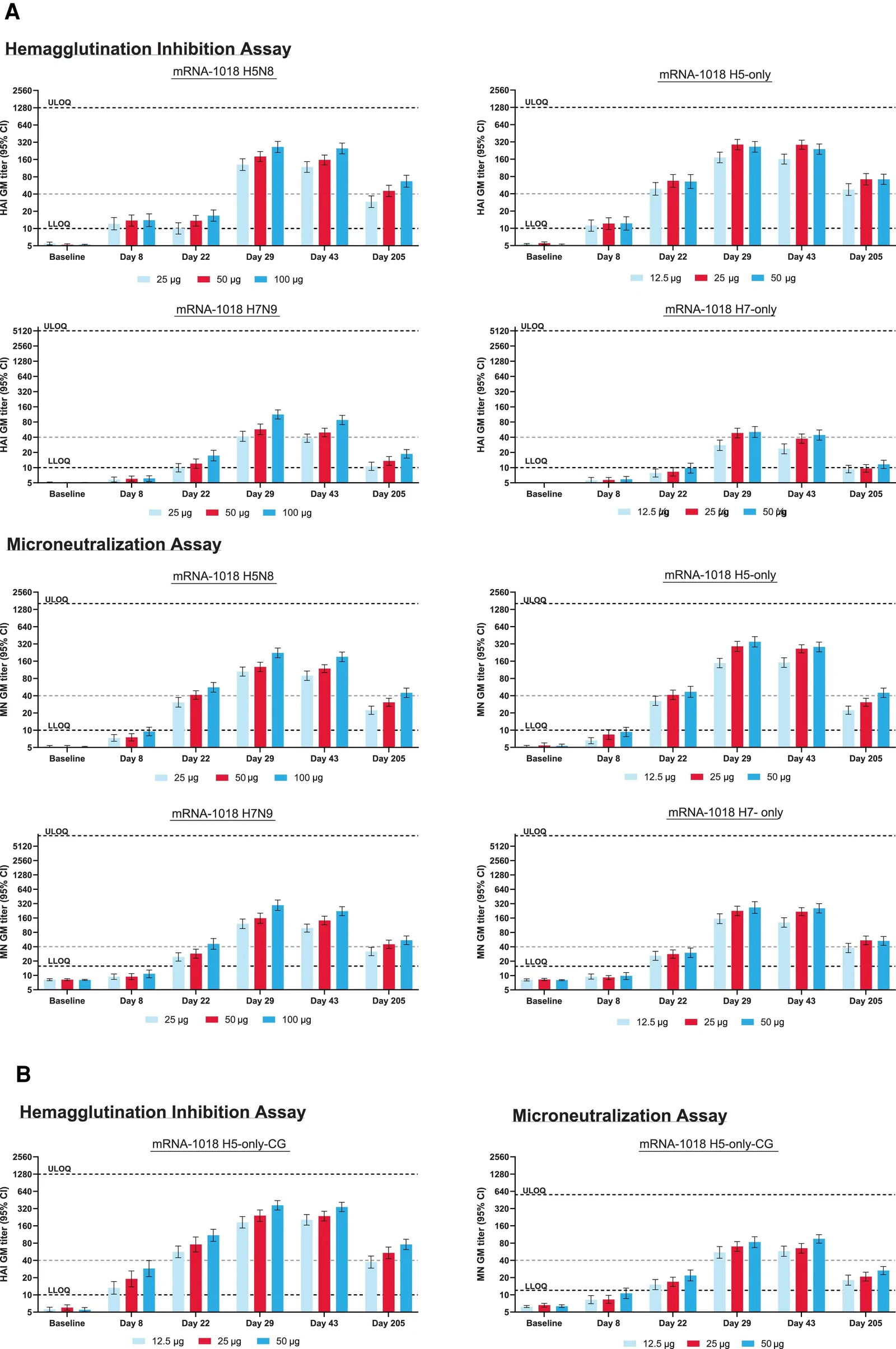
GMTs of HAI and MN antibody levels against pandemic influenza A strains after vaccination with mRNA-1018 in (*A*) part A and (*B*) part B in the overall population, PP set. The PP set consisted of all study participants who complied with the injection schedule, the timings of humoral immunogenicity blood samplings, had baseline and ≥1 evaluable postinjection assessment corresponding to the immunogenicity analysis objectives, did not have influenza infection at baseline through day 43, and had no major protocol deviations that impacted key or critical data. Participants were analyzed according to the study injection group to which they were randomized. Antibody values reported as below the LLOQ were replaced by 0.5 × LLOQ; values greater than the ULOQ were converted to the ULOQ. 95% CIs were calculated based on the *t* distribution of the log-transformed values or the difference in the log-transformed values for GMT and GMFR, respectively, and then back-transformed to the original scale for presentation. Abbreviations: CI, confidence interval; GMFR, geometric mean fold rise; GMT, geometric mean titer; HAI, hemagglutination inhibition; LLOQ, lower limit of quantification; MN, microneutralization; PP, per-protocol; ULOQ, upper limit of quantification.

**Table 2.** Immune Response by Hemagglutination Inhibition, Microneutralization, and Neuraminidase Inhibition Assays in the Overall Population, Per-Protocol Set

	mRNA-1018 Vaccine Candidate Groups by Dose Level														
	H5N8			H5-only			H7N9			H7-only			H5-only-CG		
	25 µg	50 µg	100 µg	12.5 µg	25 µg	50 µg	25 µg	50 µg	100 µg	12.5 µg	25 µg	50 µg	12.5 µg	25 µg	50 µg
	n = 94	n = 92	n = 93	n = 98	n = 91	n = 90	n = 90	n = 93	n = 94	n = 95	n = 92	n = 97	n = 95	n = 93	n = 95
Hemagglutination inhibition assay**^a^**															
Day 1, n	94	92	93	98	91	90	90	93	94	95	92	97	95	93	95
GMT	5.5	5.2	5.2	5.3	5.5	5.1	5.1	5.0	5.0	5.0	5.0	5.0	5.6	6.0	5.5
95% CI	(5.2, 5.8)	(5.0, 5.4)	(5.0, 5.3)	(5.1, 5.4)	(5.2, 5.8)	(5.0, 5.3)	(5.0, 5.2)	(5.0, 5.1)	(5.0, 5.1)	N/A	N/A	N/A	(5.1, 6.1)	(5.4, 6.7)	(5.1, 6.0)
Day 22, n	94	92	93	98	91	90	90	93	94	95	92	97	95	93	95
GMT	55.3	71.4	86.5	48.8	67.1	65.2	9.9	12.0	17.3	7.8	8.3	9.8	56.4	75.7	109.5
95% CI	(43.3, 70.6)	(57.5, 88.8)	(68.8, 108.7)	(37.8, 62.8)	(52.0, 86.6)	(49.3, 86.2)	(8.2, 12.0)	(9.7, 14.8)	(13.6, 22.1)	(6.5, 9.4)	(6.8, 10.0)	(7.8, 12.2)	(44.6, 71.2)	(56.0, 102.3)	(86.0, 139.3)
SC%	68.1	77.2	81.7	63.3	70.3	75.6	10.0	16.1	25.5	9.5	12.0	14.4	73.7	74.2	86.3
95% CI	(57.7, 77.3)	(67.2, 85.3)	(72.4, 89.0)	(52.9, 72.8)	(59.8, 79.5)	(65.4, 84.0)	(4.7, 18.1)	(9.3, 25.2)	(17.1, 35.6)	(4.4, 17.2)	(6.1, 20.4)	(8.1, 23.0)	(63.6, 82.2)	(64.1, 82.7)	(77.7, 92.5)
HAI titer ≥ 1:40, %	68.1	77.2	87.7	63.3	70.3	75.6	10	16.1	25.5	9.5	12.0	14.4	75.8	75.3	87.4
95% CI	(57.7, 77.3)	(67.2, 85.3)	(72.4, 89.0)	(52.9, 72.8)	(59.8, 79.5)	(65.4, 84.0)	(4.7, 18.1)	(9.3, 25.2)	(17.1, 35.6)	(4.4, 17.2)	(6.1, 20.4)	(8.1, 23.0)	(65.9, 84.0)	(65.2, 83.6)	(79.0, 93.3)
Day 43, n	92	91	90	93	89	86	87	92	94	93	91	90	93	92	93
GMT	118.4	157.0	249.1	160.6	285.8	239.4	38.1	49.6	87.7	23.6	37.5	44.2	203.9	236.7	340.9
95% CI	(95.3, 147.0)	(128.9, 191.2)	(200.8, 309.1)	(131.9, 195.6)	(238.0, 343.3)	(194.8, 294.3)	(31.5, 46.1)	(40.9, 60.2)	(71.2, 108.1)	(18.8, 29.5)	(30.4, 46.3)	(35.1, 55.7)	(164.7, 252.4)	(194.7, 287.8)	(283.1, 410.6)
SC%	91.3	93.4	94.4	95.7	98.9	97.7	60.9	69.6	89.4	40.9	58.2	63.3	94.6	96.7	100.0
95% CI	(83.6, 96.2)	(86.2, 97.5)	(87.5, 98.2)	(89.4, 98.8)	(93.9, 100)	(91.9, 99.7)	(49.9, 71.2)	(59.1, 78.7)	(81.3, 94.8)	(30.8, 51.5)	(47.4, 68.5)	(52.5, 73.2)	(87.9, 98.2)	(90.8, 99.3)	(96.1, 100)
HAI titer ≥ 1:40, %	91.3	93.4	94.4	95.7	98.9	97.7	60.9	69.6	89.4	40.9	58.2	63.3	95.7	97.8	100.0
95% CI	(83.6, 96.2)	(86.2, 97.5)	(87.5, 98.2)	(89.4, 98.8)	(93.9, 100))	(91.9, 99.7)	(49.9, 71.2)	(59.1, 78.7)	(81.3, 94.8)	(30.8, 51.5)	(47.4, 68.5)	(52.5, 73.2)	(89.4, 98.8)	(92.4, 99.7)	(96.1, 100)
Day 205, n	92	89	91	96	87	88	86	91	89	92	86	93	92	87	90
GMT	29.4	45.3	66.9	47.4	71.5	71.4	10.6	13.6	18.7	9.3	9.6	11.6	37.4	53.9	75.8
95% CI	(23.2, 37.2)	(36.2, 56.7)	(52.5, 85.1)	(37.5, 59.8)	(56.8, 90.1)	(58.1, 87.6)	(8.8, 12.8)	(11.1, 16.5)	(15.4, 22.7)	(7.9, 11.1)	(8.1, 11.5)	(9.6, 14.0)	(29.3, 47.6)	(42.7, 68.1)	(61.4, 93.6)
SC%	51.1	67.4	76.9	66.7	79.3	85.2	16.3	20.9	27.0	10.9	7.0	18.3	57.6	69.0	81.1
95% CI	(40.4, 61.7)	(56.7, 77.0)	(66.9, 85.1)	(56.3, 76.0)	(69.3, 87.3)	(76.1, 91.9)	(9.2, 25.8)	(13.1, 30.7)	(18.1, 37.4)	(5.3, 19.1)	(2.6, 14.6)	(11.0, 27.6)	(46.9, 67.9)	(58.1, 78.5)	(71.5, 88.6)
HAI titer ≥ 1:40, %	51.1	67.4	76.9	66.7	79.3	85.2	16.3	20.9	27.0	10.9	7.0	18.3	59.8	71.3	81.1
95% CI	(40.4, 61.7)	(56.7, 77.0)	(66.9, 85.1)	(56.3, 76.0)	(69.3, 87.3)	(76.1, 91.9)	(9.2, 25.8)	(13.1, 30.7)	(18.1, 37.4)	(5.3, 19.1)	(2.6, 14.6)	(11.0, 27.6)	(49.0, 69.9)	(60.6, 80.5)	(71.5, 88.6)
Microneutralization assay^b^															
Day 1, n	94	92	93	98	91	90	90	93	94	95	92	97	95	93	95
GMT	5.2	5.1	5.1	5.2	5.4	5.3	8.1	8.1	8.0	8.1	8.2	8.0	6.2	6.6	6.3
95% CI	(5.0, 5.4)	(4.9, 5.4)	(4.9, 5.2)	(5.0, 5.4)	(4.9, 6.0)	(4.9, 5.7)	(7.7, 8.5)	(7.7, 8.5)	(7.8, 8.2)	(7.7, 8.5)	(7.8, 8.7)	(7.8, 8.2)	(6.0, 6.5)	(6.1, 7.1)	(6.0, 6.7)
Day 22, n	94	92	93	98	91	90	90	93	94	95	92	97	95	93	95
GMT	30.7	41.2	55.9	32.4	41.2	46.6	24.4	28.5	45.6	25.8	28.1	29.8	15.1	16.9	21.8
95% CI	(25.3, 37.3)	(34.7, 48.9)	(46.0, 68.0)	(27.0, 39.0)	(34.0, 49.9)	(37.3, 58.2)	(19.9, 29.7)	(22.9, 35.4)	(35.2, 59.1)	(20.8, 31.9)	(22.9, 34.4)	(23.8, 37.5)	(12.3, 18.5)	(14.0, 20.3)	(17.5, 27.1)
≥4-fold rise	37.2	51.1	57	38.8	47.3	51.1	12.2	18.3	30.9	17.9	22.8	24.7	13.7	10.8	23.2
95% CI	(27.5, 47.8)	(40.4, 61.7)	(46.3, 67.2)	(29.1, 49.2)	(36.7, 58.0)	(40.3, 61.8)	(6.3, 20.8)	(11.0, 27.6)	(21.7, 41.2)	(10.8, 27.1)	(14.7, 32.8)	(16.5, 34.5)	(7.5, 22.3)	(5.3, 18.9)	(15.1, 32.9)
Day 43, n	92	91	90	93	89	86	87	92	94	93	91	90	93	92	93
GMT	89.4	118.7	190.3	151.1	263.0	283.0	97.9	141.4	221.6	129.2	216.7	255.1	57.7	64.9	94.8
95% CI	(73.9, 108.0)	(101.4, 138.9)	(157.1, 230.5)	(124.6, 183.2)	(223.8, 309.1)	(232.5, 344.5)	(80.5, 119.0)	(114.9, 173.9)	(178.5, 275.1)	(102.4, 163.0)	(178.9, 262.5)	(203.6, 319.7)	(47.0, 70.9)	(53.4, 78.7)	(80.0, 112.2)
≥4-fold rise	80.4	95.6	96.7	93.5	98.9	95.3	63.2	77.2	87.2	76.3	91.2	91.1	52.7	68.5	81.7
95% CI	(70.9, 88.0)	(89.1, 98.8)	(90.6, 99.3)	(86.5, 97.6)	(93.9, 100)	(88.5, 98.7)	(52.2, 73.3)	(67.2, 85.3)	(78.8, 93.2)	(66.4, 84.5)	(83.4, 96.1)	(83.2, 96.1)	(42.1, 63.1)	(58.0, 77.8)	(72.4, 89.0)
Day 205, n	92	89	91	96	87	88	86	91	89	92	86	93	92	87	90
GMT	22.3	30.7	44.8	32.1	59.2	65.5	31.6	44.7	54.3	37.7	54.4	52.9	18	20.8	26.5
95% CI	(18.9, 26.3)	(26.1, 36.1)	(37.1, 54.1)	(27.3, 37.7)	(50.4, 69.6)	(53.1, 80.7)	(25.9., 38.7)	(36.5, 54.7)	(44.1, 66.9)	(30.1, 47.2)	(44.2, 66.9)	(42.7, 65.6)	(14.7, 22.1)	(17.4, 24.8)	(22.5, 31.2)
≥4-fold rise	20.7	32.6	53.8	39.6	72.4	65.9	25.6	34.1	42.7	31.5	45.3	43.0	17.4	12.6	18.9
95% CI	(12.9, 30.4)	(23.0, 43.3)	(43.1, 64.4)	(29.7, 50.1)	(61.8, 81.5)	(55.0, 75.7)	(16.8, 36.1)	(24.5, 44.7)	(32.3, 53.6)	(22.2, 42.0)	(34.6, 56.5)	(32.8, 53.7)	(10.3, 26.7)	(6.5, 21.5)	(11.4, 28.5)
Neuraminidase inhibition assay^c^															
Day 1, n	94	92	93	-	-	-	90	93	94	-	-	-	-	-	-
GMT	6.2	5.7	7.3	-	-	-	5.5	5.3	5.4	-	-	-	-	-	-
95% CI	(5.3, 7.3)	(5.1, 6.5)	(6.0, 8.7)	-	-	-	(5.0, 6.1)	(5.0, 5.7)	(5.0, 5.8)	-	-	-	-	-	-
Day 22, n	94	92	93	-	-	-	90	93	94	-	-	-	-	-	-
GMT	17.8	34.1	49.7	-	-	-	12.8	14.7	21.5	-	-	-	-	-	-
95% CI	(14.1, 22.3)	27.5, 42.4)	(39.5, 62.3)	-	-	-	(10.2, 16.0)	(12.1, 17.9)	(17.1, 27.1)	-	-	-	-	-	-
≥4-fold rise	26.6	55.4	63.4	-	-	-	15.6	23.7	34.0	-	-	-	-	-	-
95% CI	(18.0, 36.7)	(44.7, 65.8)	(52.8, 73.2)	-	-	-	(8.8, 24.7)	(15.5, 33.6)	(24.6, 44.5)	-	-	-	-	-	-
Day 43, n	92	91	90	-	-	-	87	92	94	-	-	-	-	-	-
GMT	98.0	147.1	222.8	-	-	-	125.0	167.4	280.2	-	-	-	-	-	-
95% CI	(80.3, 119.6)	(124.8, 173.5)	(191.0, 260.0)	-	-	-	(99.3, 157.3)	(132.4, 211.7)	(228.0, 344.5)	-	-	-	-	-	-
≥4-fold rise	87.0	95.6	95.6	-	-	-	90.8	92.4	98.9	-	-	-	-	-	-
95% CI	(78.3, 93.1)	(89.1, 98.8)	(89.0, 98.8)	-	-	-	(82.7, 95.9)	(84.9, 96.9)	(94.2, 100)	-	-	-	-	-	-
Day 205, n	92	89	91	-	-	-	85	91	89	-	-	-	-	-	-
GMT	64.3	104.3	154.0	-	-	-	126.3	175.3	255.3	-	-	-	-	-	-
95% CI	(51.6, 80.1)	(85.5, 127.2)	(124.0, 191.3)	-	-	-	(101.0, 157.9)	(143.6, 214.1)	(210.5, 309.6)	-	-	-	-	-	-
≥4-fold rise	77.2	89.9	90.1	-	-	-	90.6	94.5	98.9	-	-	-	-	-	-
95% CI	(67.2, 85.3)	(81.7, 95.3)	(82.1, 95.4)	-	-	-	(82.3, 95.8)	(87.6, 98.2)	(93.9, 100)	-	-	-	-	-	-

Abbreviations: CI, confidence interval; GMT, geometric mean titer; HAI, hemagglutination inhibition assay; LLOQ, lower limit of quantification; SC, seroconversion; ULOQ, upper limit of quantification.

^a^HAI ranges (LLOQ–ULOQ) were 10–1280 for H5N8 A/Astrakhan/3212/2020, 10–5120 for H7N9 A/Anhui/1/2013, and 10–1280 for H5N1 A/chicken/Ghana/2021.

^b^Microneutralization assay ranges (LLOQ–ULOQ) were 10–1618.1 for H5N8 A/Astrakhan/3212/2020, 15.6–8445.4 for H7N9 A/Anhui/1/2013, and 12–558.9 for H5N1 A/chicken/Ghana/2021.

^c^Neuraminidase assay ranges (LLOQ–ULOQ) were 10–10 240 for H5N8 A/Astrakhan/3212/2020 and 10–20 480 for H7N9 A/Anhui/1/2013.

Regardless of the vaccine candidate tested, HAI titers were detectable prior to the booster dose on day 22, peaked on day 29 or 43, and remained detectable through day 205 across all dose levels in parts A and B (Table 2 and Figure 3). Higher HAI titers were induced with increasing vaccine dose, with the highest titers observed in participants who received vaccines containing 50 µg of HA (Figure 3). Across all dose levels, HAI titers were generally higher in participants who received H5-containing compared with H7-containing vaccine candidates (Table 2). The presence of NA in the vaccine construct did not appear to adversely impact HAI titers for the H5-containing vaccines but may have resulted in qualitatively lower titers for the H7-containing vaccines.

Functional immune responses were also assessed by MN assay. In part A, day 43 MN GMTs (95% CI) were 89.4 (73.9–108.0), 118.7 (101.4–138.9), and 190.3 (157.1–230.5) for the H5N8 25, 50, and 100 µg groups, respectively; 151.1 (124.6–183.2), 263.0 (223.8–309.1), and 283.0 (232.5–344.5) for the H5-only 12.5, 25, and 50 µg groups, respectively; 97.9 (80.5–119.0), 141.4 (114.9–173.9), and 221.6 (178.5–275.1) for the H7N9 25, 50, and 100 µg groups, respectively; and 129.2 (102.4–163.0), 216.7 (178.9–262.5), and 255.1 (203.6, 319.7) for the H7-only 12.5, 25, and 50 µg groups, respectively (Table 2). In part B, day 43 MN GMTs (95% CI) were 57.7 (47.0–70.9), 64.9 (53.4–78.7), and 94.8 (80.0–112.2) in the H5-only-CG 12.5, 25, and 50 µg groups, respectively. A ≥4-fold rise in MN titers at day 43 relative to baseline occurred in 80.4%–96.7% of participants in the H5N8 group, 93.5%–98.9% of those in the H5-only group, 63.2%–87.2% of those in the H7N9 group, 76.3%–91.2% of those in the H7-only group, and 52.7%–81.7% of those in the H5-only-CG group. In contrast to the results of the HAI assay, immune responses assessed by MN were similar between the H5- and H7-containing vaccines (Figure 3).

On day 43, assessment by NAI revealed GMTs (95% CI) of 98.0 (80.3–119.6), 147.1 (124.8–173.5), and 222.8 (191.0–260) in the H5N8 25, 50, and 100 µg groups, respectively, and 125.0 (99.3–157.3), 167.4 (132.4–211.7), and 280.2 (228.0–344.5) in the respective H7N9 groups (Table 2). Titers were detectable on day 22 across dose levels for the H5N8 and H7N9 vaccines, and then notably increased, peaking on day 43 (Table 2). While NAI responses appeared to decrease after day 43, the percentage of participants with a ≥4-fold rise compared with baseline ranged between 77.2% and 98.9% across all dose levels for both vaccines.

The H5-containing vaccine elicited robust HAI GMTs in both studied age groups (18–64 and ≥65 years) across all time points (Supplementary Table 4). Among participants receiving H7-containing constructs, adults 18–64 years had higher peak HAI GMTs compared with those ≥65 years across dose levels. Microneutralization responses in the 2 age groups at day 43 were similar to those in the overall group. Neuraminidase inhibition responses tended to be higher in those 18–64 years compared with those ≥65 years.

## DISCUSSION

This phase 1/2 study evaluated the safety, reactogenicity, and immunogenicity of 5 mRNA-1018 candidates targeting 2 different influenza A virus groups with pandemic potential (H5N8/H5N1 and H7N9) in adults ≥ 18 years. The candidates had acceptable safety and tolerability profiles across dose levels with no vaccine-related safety concerns identified. Immune responses against homologous influenza strains, assessed by a variety of standard functional assays, including HAI, MN, and NAI, were strong and sustained and increased with mRNA-1018 vaccine dose.

All mRNA-1018 candidates were generally well tolerated without clinically significant safety concerns through 6 months across the range of dose levels tested. Most solicited ARs were grade 1–2 in severity, transient, and self-limiting. A dose-dependent increase in the incidence of solicited ARs was observed, with the highest incidence reported for the H5N8 100 µg group. Few participants reported AEs that resulted in vaccine discontinuation, and only 1 case was assessed as vaccine related. Solicited local and systemic ARs occurred more frequently among participants 18–64 years versus those ≥65 years in part A, while incidences were similar between age subgroups in part B.

All mRNA-1018 vaccine candidates tested induced strong functional immune responses as measured by orthologous assays using pandemic virus strains homologous to the vaccine candidates. Immune responses were detectable prior to the second dose of vaccine, peaking either 1 or 3 weeks after the second dose and persisting for 6 months after the last dose. The H5-based candidates (H5N8, H5-only, and H5-only-CG) induced stronger strain-specific immune responses by HAI titer compared with the H7-based candidates. Responses increased with mRNA dose for each candidate vaccine. Additionally, the inclusion of NA antigens N8 and N9 in the H5- and H7-encoding mRNA-1018 vaccines did not appear to negatively impact HAI responses. The results of MN testing were generally consistent with the results obtained by HAI assay across vaccine candidates. Notably, while HAI titers against the H5 strains were mostly higher compared with those against the H7 strains, MN titers were similar between the H5- and H7-based candidates.

The results of this study are further supported by results from the phase 3 study of mRNA-1010, a trivalent seasonal influenza vaccine designed as an HA-only vaccine and manufactured using the same platform as mRNA-1018. This clinical endpoint study demonstrated superior efficacy of mRNA-1010 for preventing influenza versus a licensed comparator [18].

The present findings compare favorably with data supporting the licensure of approved H5N1 pandemic influenza vaccines. Inactivated influenza vaccines derived from H5 and H7 influenza strains are poorly immunogenic in the absence of adjuvant [24–29]. H7 strains do not induce strong HAI responses and neutralization even when combined with adjuvants, including MF59 and ASO3 [30, 31]. In a phase 3 study in adults ≥ 18 years (NCT00616928 [32]), a licensed, inactivated, split-virion, AS03-adjuvanted vaccine (GlaxoSmithKline) achieved an HAI ≥ 1:40 titer in 90.8% of participants 18–64 years and 74.5% of participants ≥ 65 years [33]. Similarly, a licensed, inactivated, MF59-adjuvanted subunit vaccine (Seqirus, Inc.) achieved an HAI titer of ≥1:40 on day 43 in 80%–83% of participants 18–64 years and in 54%–74% of participants ≥ 65 years [34].

This phase 1/2 study was relatively large, providing greater confidence in the assay results, which have inherent variability as biologic assays. The study included adults across a wide range of ages, contributing to the generalizability of the results and mitigating potential sources of immunologic variability due to past exposure to seasonal and pandemic influenza strains. The vaccine candidates were clinically relevant, particularly since the sequences of both H5 strains were based on candidate vaccine viruses from clade 2.3.4.4b, the same as the HPAI strains causing the ongoing outbreaks in cattle and recent human disease [35]. An important limitation is that while the assays were qualified for use in clinical studies, they were not validated, an expectation for a future pivotal study.

In conclusion, the findings from this phase 1/2 study demonstrated that the 5 tested mRNA-1018 candidates were generally well tolerated across all dose levels tested and had an acceptable safety profile. These vaccine candidates induced strong and durable neutralizing immune responses to vaccine-matched influenza strains across all dose levels tested and for different age subgroups. These results combined with the demonstrated efficacy of mRNA-1010 support further development of mRNA-1018 as a promising approach for pandemic preparedness.

## Supplementary Material

ciag278_Supplementary_Data

## Supplementary Data

Supplementary materials are available at *Clinical Infectious Diseases* online. Consisting of data provided by the authors to benefit the reader, the posted materials are not copyedited and are the sole responsibility of the authors, so questions or comments should be addressed to the corresponding author.
