# Supplementary material for: A Phase 1/2 Dose-Ranging Safety and Immunogenicity Study of mRNA-Based Candidate Pandemic Influenza Vaccines in Healthy Adults
Source: Clin Infect Dis. 2026 Apr 25;83(3):e598–610. doi: 10.1093/cid/ciag278 (PMC13644865; doi:10.1093/cid/ciag278)
Supplement: Supplementary file 1 [file ciag278_supplementary_data.docx]

Supplementary Appendix

**A Phase 1/2 Dose-Ranging Safety and Immunogenicity Study of mRNA-Based Candidate Pandemic Influenza Vaccines in Healthy Adults**

This appendix has been provided by the authors to give readers additional information.

# Supplementary Methods

## Inclusion and Exclusion Criteria

Each participant was required to have met the following criteria for enrollment into the study:

- An adult, ≥18 years of age at the time of informed consent, who was healthy based on review of medical history, physical examination, laboratory tests, and results of electrocardiogram testing
- Understood and was willing and physically able to comply with protocol-mandated follow-up, including all procedures, in the opinion of the Investigator
- Had a body mass index of 18 kg/m^2^ to 35 kg/m^2^ (inclusive) at screening
- If assigned female at birth and not pregnant or breast/chest/body feeding, could be enrolled in the study if one of the following applied:
  - Not a person of childbearing potential (a person of childbearing potential was defined as following menarche until becoming postmenopausal or becoming permanently sterile) or
  - A person of childbearing potential and using a permitted contraceptive method (intrauterine device; intrauterine hormone-releasing system; bilateral tubal occlusion; progestogen-only hormone contraception; combined estrogen- and progestogen-containing hormonal contraception; external or internal condom; cervical cap, diaphragm, or sponge with spermicide; combination of external condom with either cervical cap, diaphragm, or sponge with spermicide; azoospermic partner; sexual abstinence) during the study and for ≥3 months after the last dose of study vaccine

Participants who met any of the following criteria were excluded from the study:

- Was acutely ill or febrile (body temperature ≥38.0°C/100.4°F) 72 hours prior to or at screening, or day 1. Participants meeting this criterion could be rescheduled within the 28-day screening window and would retain their initially assigned participant number
- Had a preexisting diagnosis or condition that was clinically unstable, or that could have affected participant safety, adherence to study procedures, or the assessment of vaccine safety or immunogenicity, as determined by the Investigator. Clinically unstable was defined as a diagnosis or condition requiring significant changes in management or medication ≤60 days prior to screening and included ongoing workup of an undiagnosed illness that could lead to a new diagnosis or condition
- Had a history of myocarditis, pericarditis, or myopericarditis
- Had a history of congenital or acquired immunodeficiency, immunosuppressive condition, or immune-mediated disease
- Had any dermatologic conditions that could affect local solicited adverse reaction (AR) assessments (eg, tattoos, psoriasis patches affecting skin over the deltoid areas)
- Had a history of anaphylaxis or severe hypersensitivity reaction after receipt of any mRNA-containing vaccine or the components contained in the investigational vaccine
- Had a history of bleeding disorder considered a contraindication to intramuscular injection or phlebotomy
- Had a medical, psychiatric, or occupational condition, including history of substance abuse, that could pose additional risk as a result of participation or that could interfere with interpretation of study results according to the Investigator’s judgment
- Had received systemic immunosuppressants or immune-modifying drugs for >14 days in total within 180 days prior to screening (for corticosteroids ≥10 mg/day of prednisone or equivalent) or was anticipating the need for immunosuppressive treatment at any time during participation in the study
- Had a screening laboratory value (complete blood count, alanine aminotransferase, aspartate aminotransferase, creatinine, alkaline phosphatase, or total bilirubin) abnormality grade >1 in severity
- Had received or planned to receive any vaccine authorized or approved by a local health agency (including mRNA vaccine) ≤28 days prior to the vaccination (day 1), or had planned to receive any such vaccine within 28 days after vaccination
- Had received systemic immunoglobulins or blood products within 90 days prior to screening or had plans to receive them during the study
- Had a diagnosis of malignancy within the previous 10 years (excluding nonmelanoma skin cancer)
- Had donated ≥450 mL of blood products within 28 days prior to screening or planned to donate blood products during the study
- Had participated in an interventional clinical study within 28 days prior to screening based on the medical history interview or planned to do so while participating in this study
- Was an immediate family member or household member of study personnel, study site staff, or Sponsor personnel

## Randomization and Blinding

Randomization was conducted separately for Parts A and B. Participants were aged-stratified in both study parts (18–64, or ≥65 years), with an enrollment of ≥30% of the total participants in either age group. Participants were randomly assigned to vaccine by a central interactive voice response system/interactive Web-response system. Separate randomization lists were maintained for Parts A and B.

This was an observer-blind study, with the Investigator, study staff, study participants, site monitors, and Sponsor personnel all blinded to the vaccine administered until the study database was locked and unblinded. A limited number of unblinded personnel were assigned to vaccine accountability procedures and prepared the study vaccines, while an unblinded medically qualified study site personnel administered the vaccinations. Unblinded site monitors were assigned responsibilities to ensure sites were following all proper vaccination accountability, preparation, and administration procedures.

## Vaccine Strains

Strain selection for mRNA-1018 candidate vaccines was based on World Health Organization (WHO) zoonotic influenza A candidate vaccine viruses issued in September 2024 [1-3]. In Part B of the study, an A(H5N1) strain, referred to as H5-only CG, was selected to directly address concerns about the emergence of highly pathogenic avian A(H5N1) strains. The H5-only-CG vaccine candidate encodes H5 hemagglutinin of A/chicken/Ghana/2021 strain, which is closely related to clade 2.3.4.4b (genotype B3.13) viruses which have been detected in Texas dairy cattle and were the cause of human infections in early 2024 [4, 5]. The A/chicken/Ghana/2021 strain is currently being prepared as a candidate vaccine virus by the WHO [3].

## Hemagglutination Inhibition Assay

Hemagglutination (HA) inhibition (HAI) assays were performed using horse red blood cells in accordance with a qualified Good Clinical Laboratory Practice (GCLP)-compliant method recommended for the assessment of antibody responses to pandemic influenza viruses. The HAI method used was based on the method developed and recommended by the WHO [6].

Prior to analysis, serum samples were heat-inactivated and adsorbed with horse red blood cells before being treated with receptor-destroying enzyme to remove non-specific agglutinins and inhibitors. Treated sera were tested in 2-fold serial dilutions against standardized influenza virus antigen propagated in myeloid dendritic cells (MDCs) and calibrated to 4 HA units, followed by the addition of 1% horse red blood cells. After incubation at room temperature, inhibition of HA was determined by visual inspection. HAI titers were defined as the reciprocal of the highest serum dilution that completely inhibited HA, consistent with regulatory guidance. Assay validity was confirmed using virus back titration and appropriate positive and negative control sera, and all samples were tested in duplicate and independently read using predefined acceptability criteria.

HAI assays were performed at VisMederi (Siena, Italy), with sample testing conducted in the first half of 2025. Samples from the same participant were tested within the same run whenever possible. Quality control (QC) samples were included in each run, and runs falling outside the predefined QC acceptance criteria were repeated. QC acceptance ranges were established during assay qualification and were defined as the median QC titer based on repeated measurements, ± one titer step.

## Neuraminidase Inhibition Assay

Neuraminidase (NA)-inhibiting (NAI) antibody responses were measured using an enzyme-linked lectin assay employing strain-specific recombinant NA from influenza, which was based on previously published methodology [7-9]. Microplates were coated with fetuin, and heat-inactivated human serum samples were serially diluted and incubated with NA at a predetermined EC_70_ working concentration to enable sensitive detection of inhibition. Following overnight incubation, NA activity was quantified using horseradish peroxidase (HRP)-conjugated peanut agglutinin and a chromogenic substrate, with optical density measured at 450 nm. Percent inhibition was calculated relative to NA-only control wells after subtraction of plate blanks. NAI titers were defined as the reciprocal of the highest serum dilution achieving at least 50% inhibition of NA activity. Each assay run included NA maximum activity controls, assay blanks, and positive and negative control sera to verify assay performance. Samples were tested in singlicate as defined by assay qualification, and the results were evaluated using predefined acceptance criteria.

NAI assays were performed at IQVIA (Laval), with sample testing conducted in the first half of 2024. QC samples were included in each run, and runs falling outside the predefined QC acceptance criteria were repeated. QC acceptance ranges were established during assay qualification and were defined as the median QC titer based on repeated measurements, ± one titer step.

## Microneutralization Assay

An enzyme-linked immunosorbent assay (ELISA)-based single-cycle microneutralization (MN) assay was performed using live influenza virus and Madin-Darby canine kidney (MDCK) cells to quantify functional neutralizing antibody responses against pandemic influenza strains. The method used was based on the method developed and recommended by the WHO [10]. Serum samples were heat-inactivated prior to testing. Two-fold serial dilutions of serum were incubated with a standardized virus that was propagated in MDCK cells and calibrated to 100 × the 50% tissue culture infectious dose per well, followed by infection of MDCK cell monolayers. After incubation, cells were fixed and viral replication was quantified by ELISA detection of influenza nucleoprotein using primary antibodies and HRP-conjugated secondary antibodies. Neutralization was defined as inhibition of viral infection relative to virus control wells, with neutralizing titers calculated as the serum dilution corresponding to 50% reduction in infection using linear interpolation. Virus back-titration, cell controls, virus controls, and positive and negative control sera were included in each assay run to confirm assay run data validity. All samples were tested in duplicate, with visits from sample participants batched in the same assay run. Results were evaluated using predefined acceptability and retesting criteria.

MN assays were performed at VisMederi (Siena, Italy), with sample testing conducted in the first half of 2025. Samples from the same participant were tested within the same run whenever possible. QC samples were included in each run, and runs falling outside the predefined QC acceptance criteria were repeated. QC acceptance ranges were established during assay qualification and were defined as the median QC titer based on repeated measurements, ± one titer step.

## Sample Size

Sample sizes for both parts of the study were not based on formal statistical hypothesis testing. It was expected that with 100 participants in each vaccine candidate group, there was an approximately 95% probability to observe at least 1 participant with an adverse event (AE) if the true incidence rate of AEs was 3%.

Study Populations

Immunogenicity analyses were performed in the per-protocol population, which included all randomly assigned participants who complied with the vaccination schedule, complied with baseline and ≥1 post-injection time point blood sampling, did not have influenza infection at baseline through day 43, and had no major protocol deviation that impacted the immune response. All safety assessments except for solicited local and systemic ARs were assessed in the safety population, which included all randomized participants who received vaccination. The solicited safety set consisted of all participants in the safety set who reported any solicited AR event.

Safety Analyses

Participants used an electronic diary to record local ARs (ie, injection site pain, injection site redness, injection site hardness, or axillary swelling/tenderness ipsilateral to the side of injection), or systemic ARs (ie, fever, headache, fatigue, myalgia, arthralgia, nausea/vomiting, or chills). The number and percentage of participants with any solicited local AR or solicited systemic AR during the 7-day follow-up period after each investigational medicinal product (IMP) injection were summarized. The 2-sided 95% CI calculated using the Clopper-Pearson method was also provided for the percentage of participants with any solicited AR after each IMP injection. The number of events of unsolicited AEs, serious AEs, AE of special interest, medically attended AEs, and AEs leading to study withdrawal were summarized, while descriptive summary statistics were provided for all other safety analyses.

Immunogenicity Analyses

Geometric mean titers (GMT) with corresponding 95% CIs and geometric mean fold rises (GMFR) over day 1 (baseline) with corresponding 95% CIs were calculated for each vaccination group; 95% CIs were calculated based on the *t* distribution of the log-transformed values or the difference in the log-transformed values for GMT and GMFR, respectively, and then back-transformed to the original scale. The HAI seroconversion rate from baseline was determined along with 2-sided 95% CIs (calculated using the Clopper-Pearson method). The rate of HAI seroconversion was defined as the percentage of participants with either a pre-vaccination HAI titer <1:10 and a post-vaccination HAI titer ≥1:40 or a pre-vaccination HAI titer ≥1:10 and a minimum 4-fold rise in post-vaccination HAI antibody titer. The percentage of participants with ≥2-, 3-, or 4-fold rise from day 1 in NAI titers was determined along with 2-sided 95% CIs (calculated using the Clopper-Pearson method). The percentage of participants with ≥2-, 3-, or 4-fold rise from day 1 in NAI titers was defined as ≥2-, 3-, or 4-fold rise of the lower limit of quantification (LLOQ) if the day 1 titer was <LLOQ, or ≥2-, 3-, or 4-fold rise of the day 1 titer if the day 1 titer was ≥LLOQ. For summarization of GMTs, antibody titers reported <LLOQ were replaced by 0.5 × LLOQ

# Supplementary Figures

Supplementary Figure 1. Overview of the study design.

CG, chicken Ghana; HA, hemagglutinin; NA, neuraminidase.

## Supplementary Figure 2. Percentages of patients with individual solicited local adverse reactions and solicited systemic adverse reactions reported within 7 days after the first or second mRNA-1018 injection by vaccine candidate (across dose levels) and severity, in (A) Part A and (B) Part B; solicited safety set.

AR, adverse reaction.

## Supplementary Figure 3. Percentages of participants reporting solicited local and systemic adverse reactions within 7 days after the first or second mRNA-1018 injection in each vaccine candidate arm by age group in (A) Part A and (B) Part B; solicited safety set. The solicited safety set included all randomized participants who received the study injection and contributed any solicited adverse reaction data.

AR, adverse reaction.

# Supplementary Tables

## Supplementary Table 1. Classification of Solicited Adverse Reactions by Grade^a^.

| **Reaction** | **Grade 0 (None)** | **Grade 1 (Mild)** | **Grade 2 (Moderate)** | **Grade 3 (Severe)** | **Grade 4 (Life-threatening)** |
| --- | --- | --- | --- | --- | --- |
| **Local** | | | | | |
| **Injection site pain** | None | No interference with activity | Some interference with activity | Prevents daily activity | Requires ER visit or hospitalization |
| **Injection site erythema (redness)** | <25 mm/  <2.5 cm | 25-50 mm/  2.5-5 cm | 51-100 mm/  5.1-10 cm | >100 mm/  >10 cm | Necrosis or exfoliative dermatitis |
| **Injection site swelling/induration (hardness)** | <25 mm/  <2.5 cm | 25-50 mm/  2.5-5 cm | 51-100 mm/  5.1-10 cm | >100 mm/  >10 cm | Necrosis |
| **Axillary (underarm) swelling or tenderness ipsilateral to the side of injection** | None | No interference with activity | Some interference with activity | Prevents daily activity | ER visit or hospitalization |
| **Systemic** | | | | | |
| **Headache** | None | No interference with activity | Some interference with activity | Prevents daily activity | Requires ER visit or hospitalization |
| **Fatigue** | None | No interference with activity | Some interference with activity | Significant; prevents daily activity | Requires ER visit or hospitalization |
| **Myalgia (muscle aches all over body)** | None | No interference with activity | Some interference with activity | Significant; prevents daily activity | Requires ER visit or hospitalization |
| **Arthralgia (joint aches in several joints)** | None | No interference with activity | Some interference with activity | Significant; prevents daily activity | Requires ER visit or hospitalization |
| **Nausea/vomiting** | None | No interference with activity or 1-2 episodes/24 hours | Some interference with activity or >2 episodes/24 hours | Prevents daily activity, requires outpatient intravenous hydration | Requires ER visit or hospitalization for hypotensive shock |
| **Chills** | None | No interference with activity | Some interference with activity not requiring medical intervention | Prevents daily activity and requires medical intervention | Requires ER visit or hospitalization |
| **Fever (oral)** | <38.0°C  <100.4°F | 38.0-38.4°C  100.4-101.1°F | 38.5-38.9°C  101.2-102.0°F | 39.0-40.0°C  102.1-104.0°F | >40.0°C  >104.0°F |

Abbreviations: C, centigrade; ER, emergency room; F, Fahrenheit.

^a^Modified from the Toxicity Grading Scale for Healthy Adult and Adolescent Volunteers Enrolled in Preventative Vaccine

Clinical Trials [11].

## Supplementary Table 2. Summary of Unsolicited AEs Throughout the Study by the Total Vaccine Group for the Overall Population, Safety Set

|  | Part A | | | | Part B | |
| --- | --- | --- | --- | --- | --- | --- |
|  | H5N8   N = 296 | H5-only N = 300 | H7N9 N = 296 | H7-only N = 303 | | H5-only-CG N = 304 |
| Unsolicited AEs regardless of relationship to study injection, n (%) | | | | | | |
| Any | 138 (46.6) | 136 (45.3) | 148 (50.0) | 141 (46.5) | | 97 (31.9) |
| Serious | 6 (2.0) | 6 (2.0) | 7 (2.4) | 5 (1.7) | | 4 (1.3) |
| Fatal | 0 | 0 | 0 | 1 (0.3) | | 0 |
| Medically attended | 95 (32.1) | 98 (32.7) | 101 (34.1) | 87 (28.7) | | 70 (23.0) |
| Leading to discontinuation from study vaccine | 3 (1.0) | 5 (1.7) | 1 (0.3) | 1 (0.3) | | 0 |
| Leading to study withdrawal | 0 | 0 | 1 (0.3) | 0 | | 0 |
| Severe (grade ≥3) | 5 (1.7) | 3 (1.0) | 6 (2.0) | 5 (1.7) | | 7 (2.3) |
| Nonserious | 132 (44.6) | 130 (43.3) | 141 (47.6) | 136 (44.9) | | 93 (30.6) |
| Severe (grade ≥3) | 1 (0.3) | 0 | 1 (0.3) | 1 (0.3) | | 3 (1.0) |
| Any AESI per Investigator | 2 (0.7) | 3 (1.0) | 0 | 2 (0.7) | | 5 (1.6) |
| Unsolicited AEs related to study injection, n (%) | | | | | | |
| Any | 14 (4.7) | 13 (4.3) | 15 (5.1) | 17 (5.6) | | 7 (2.3) |
| Serious | 0 | 0 | 0 | 0 | | 1 (0.3) |
| Fatal | 0 | 0 | 0 | 0 | | 0 |
| Medically attended | 1 (0.3) | 4 (1.3) | 1 (0.3) | 1 (0.3) | | 4 (1.3) |
| Leading to discontinuation from study vaccine | 0 | 1 (0.3) | 0 | 0 | | 0 |
| Leading to study withdrawal | 0 | 0 | 0 | 0 | | 0 |
| Severe (grade ≥3) | 0 | 0 | 0 | 0 | | 1 (0.3) |
| Nonserious | 14 (4.7) | 13 (4.3) | 15 (5.1) | 17 (5.6) | | 6 (2.0) |
| Severe (grade ≥3) | 0 | 0 | 0 | 0 | | 0 |
| Any AESI per Investigator | 0 | 1 (0.3) | 0 | 0 | | 1 (0.3) |

Abbreviations: AE, adverse event; AESI, adverse event of special interest.

## Supplementary Table 3. Summary of Unsolicited AEs Through 21 Days After the First or Second Injection by Vaccine Candidate Group (All Dose Levels Combined) for the Overall Population, Safety Set

|  | **Part A** | | | | **Part B** |
| --- | --- | --- | --- | --- | --- |
|  | H5N8 (N = 296) | H5-only (N = 300) | H7N9 (N = 296) | H7-only (N = 303) | H5-only-CG (N = 304) |
| **Unsolicited AEs within 21 days** | | | | | |
| **Regardless of relationship to study injection, n (%)** | | | | | |
| Any AE | 85 (28.7) | 88 (29.3) | 91 (30.7) | 103 (34.0) | 57 (18.8) |
| Serious | 2 (0.7) | 0 | 1 (0.3) | 1 (0.3) | 2 (0.7) |
| Fatal | 0 | 0 | 0 | 0 | 0 |
| Medically attended | 29 (9.8) | 34 (11.3) | 31 (10.5) | 26 (8.6) | 21 (6.9) |
| Leading to study vaccine discontinuation | 2 (0.7) | 4 (1.3) | 1 (0.3) | 0 | 0 |
| Leading to study withdrawal | 0 | 0 | 0 | 0 | 0 |
| Severe (grade ≥3) | 1 (0.3) | 1 (0.3) | 1 (0.3) | 1 (0.3) | 4 (1.3) |
| Non-serious^a^ | 83 (28.0) | 88 (29.3) | 90 (30.4) | 102 (33.7) | 55 (18.1) |
| Severe (grade ≥3) | 0 | 1 (0.3) | 0 | 1 (0.3) | 2 (0.7) |
| AESI | 1 (0.3) | 0 | 0 | 2 (0.7) | 5 (1.6) |
| **Related to study vaccination, n (%)** | | | | | |
| Any AE | 14 (4.7) | 12 (4.0) | 13 (4.4) | 56 (4.7) | 7 (2.3) |
| Serious | 0 | 0 | 0 | 0 | 1 (0.3) |
| Fatal | 0 | 0 | 0 | 0 | 0 |
| Medically attended | 1 (0.3) | 4 (1.3) | 1 (0.3) | 7 (0.6) | 4 (1.3) |
| Leading to study vaccine discontinuation | 0 | 1 (0.3) | 0 | 1 (<0.1) | 0 |
| Leading to study withdrawal | 0 | 0 | 0 | 0 | 0 |
| Severe (grade ≥3) | 0 | 0 | 0 | 0 | 1 (0.3) |
| Non-serious^a^ | 14 (4.7) | 12 (4.0) | 13 (4.4) | 56 (4.7) | 6 (2.0) |
| Severe (grade ≥3) | 0 | 0 | 0 | 0 | 0 |
| AESI | 0 | 0 | 0 | 0 | 1 (0.3) |

Abbreviations: AE, adverse event; AESI, adverse event of special interest.

^a^Participants who did not report any serious AE were included as “non-serious.”

## Supplementary Table 4. Immune Response by Inhibition, Neuraminidase Inhibition, and Microneutralization Antibody Assays by Age Group, Per-Protocol Set

| **18–64 years** | | | | | | **≥65 years** | | | | |
| --- | --- | --- | --- | --- | --- | --- | --- | --- | --- | --- |
|  | **Part A** | | | | **Part B** | **Part A** | | | | **Part B** |
| **Time Point** | H5N8 N = 191 | H5-only N = 189 | H7N9 N = 190 | H7-only N = 196 | H5-only-CG N = 194 | H5N8 N = 88 | H5-only N = 90 | H7N9 N = 87 | H7-only N = 88 | H5-only-CG N = 89 |
| **Hemagglutination inhibition assay^a^** | | | | | | | | | | |
| Day 1 (baseline), n | 191 | 189 | 190 | 196 | 194 | 88 | 90 | 87 | 88 | 89 |
| GMT (95% CI)^b^ | 5.2 (5.1, 5.3) | 5.2 (5.1, 5.3) | 5.1 (5.0, 5.1) | 5.0 (NA) | 5.7 (5.4, 6.1) | 5.6 (5.3, 5.9) | 5.5 (5.3, 5.8) | 5.0 (5.0, 5.1) | 5.0 (NA) | 5.6 (5.2, 6.2) |
| Day 8, n | 190 | 189 | 189 | 194 | 193 | 87 | 90 | 86 | 88 | 89 |
| GMT (95% CI)^b^ | 12.5 (10.6, 14.7) | 10.4 (8.9, 12.3) | 6.2 (5.6, 6.7) | 5.8 (5.3, 6.3) | 19.1 (15.4, 23.6) | 15.1 (11.6, 19.7) | 15.1 (11.7, 19.6) | 5.6 (5.1, 6.1) | 5.7 (5.1, 6.3) | 19.9 (14.6, 27.2) |
| HAI titer ≥1:40,^c^ % (95% CI)^d^ | 22.6 (16.9, 29.2) | 21.2 (15.6, 27.7) | 5.8 (2.9, 10.2) | 4.1 (1.8, 8.0) | 34.7 (28.0, 41.9) | 25.3 (16.6, 19.7) | 30.0 (20.8, 40.6) | 2.3 (0.3, 8.1) | 3.4 (0.7, 9.6) | 37.1 (27.1, 48.0) |
| GMFR (95% CI)^b^ | 2.4 (2.1, 2.8) | 2.0 (1.7, 2.4) | 1.2 (1.1, 1.3) | 1.2 (1.1, 1.3) | 3.3 (2.7, 4.1) | 2.7 (2.1, 3.5) | 2.7 (2.1, 3.5) | 1.1 (1.0, 1.2) | 1.1 (1.0, 1.3) | 3.5 (2.6, 4.8) |
| Seroconversion,^e^ % (95% CI)^d^ | 22.6 (16.9, 29.2) | 21.2 (15.6, 27.7) | 5.8 (2.9, 10.2) | 4.1 (1.8, 8.0) | 32.6 (26.1, 39.7) | 25.3 (16.6, 35.7) | 30.0 (20.8, 40.6) | 2.3 (0.3, 8.1) | 3.4 (0.7, 9.6) | 34.8 (25.0, 45.7) |
| Day 22, n | 191 | 189 | 190 | 196 | 194 | 88 | 90 | 87 | 88 | 89 |
| GMT (95% CI)^b^ | 72.4 (61.9, 84.6) | 58.4 (48.6, 70.1) | 13.6 (11.6, 16.0) | 9.0 (7.8, 10.3) | 86.1 (71.9, 103.0) | 64.7 (50.0, 83.6) | 61.8 (47.2, 80.9) | 11.0 (9.0, 13.6) | 7.8 (6.3, 9.6) | 61.9 (46.7, 81.9) |
| HAI titer ≥1:40,^c^ % (95% CI)^d^ | 79.6 (73.2, 85.1) | 68.8 (61.7, 75.3) | 17.9 (12.7, 24.1) | 13.3 (8.9, 18.8) | 82.0 (75.8, 87.1) | 67.0 (56.2, 76.7) | 71.1 (60.6, 80.2) | 16.1 (9.1, 25.5) | 9.1 (4.0, 17.1) | 74.2 (63.8, 82.9) |
| GMFR (95% CI)^b^ | 14.0 (12.0, 16.3) | 11.3 (9.4, 13.5) | 2.7 (2.3, 3.2) | 1.8 (1.6, 2.1) | 15.0 (12.6, 17.9) | 11.6 (9.1, 14.8) | 11.2 (8.6, 14.5) | 2.2 (1.8, 2.7) | 1.6 (1.3, 1.9) | 11.0 (8.3, 14.5) |
| Seroconversion,^e^ % (95% CI)^d^ | 79.6 (73.2, 85.1) | 68.8 (61.7, 75.3) | 17.9 (12.7, 24.1) | 13.3 (8.9, 18.8) | 81.4 (75.2, 86.7) | 67.0 (56.2, 76.7) | 71.1 (60.6, 80.2) | 16.1 (9.1, 25.5) | 9.1 (4.0, 17.1) | 70.8 (60.2, 79.9) |
| Day 29, n | 185 | 183 | 188 | 188 | 189 | 87 | 88 | 82 | 88 | 87 |
| GMT (95% CI)^b^ | 187.3 (159.4, 220.0) | 245.9 (212.1, 285.1) | 69.5 (59.1, 81.8) | 44.0 (37.1, 52.3) | 290.4 (248.4, 339.4) | 176.8 (141.0, 221.5) | 205.9 (163.6, 259.0) | 56.1 (43.0, 73.1) | 34.3 (27.3, 43.1) | 189.9 (152.5, 236.5) |
| HAI titer ≥1:40,^c^ % (95% CI)^d^ | 93.0 (88.3, 96.2) | 95.6 (91.6, 98.1) | 77.1 (70.5, 82.9) | 64.9 (57.6, 71.7) | 95.2 (91.2, 97.8) | 93.1 (85.6, 97.4) | 97.7 (92.0, 99.7) | 73.2 (62.2, 82.4) | 52.3 (41.4, 63.0) | 95.4 (88.6, 98.7) |
| GMFR (95% CI)^b^ | 36.4 (30.9, 42.7) | 47.5 (41.0, 55.1) | 13.8 (11.7, 16.2) | 8.8 (7.4. 10.5) | 51.6 (44.0, 60.4) | 31.9 (25.6, 39.7) | 37.5 (30.2, 46.5) | 11.1 (8.5, 14.5) | 6.9 (5.5, 8.6) | 33.6 (26.4, 42.6) |
| Seroconversion,^e^ % (95% CI)^d^ | 93.0 (88.3, 96.2) | 95.6 (91.6, 98.1) | 77.1 (70.5, 82.9) | 64.9 (57.6, 71.7) | 95.2 (91.2, 97.8) | 93.1 (85.6, 97.4) | 97.7 (92.0, 99.7) | 73.2 (62.2, 82.4) | 52.3 (41.4, 63.0) | 95.4 (88.6, 98.7) |
| Day 43, n | 186 | 178 | 188 | 187 | 189 | 87 | 90 | 85 | 87 | 89 |
| GMT (95% CI)^b^ | 162.4 (138.8, 190.1) | 226.7 (198.5, 259.0) | 56.8 (49.3, 65.3) | 35.2 (30.1, 41.2) | 278.4 (241.9, 320.4) | 174.7 (142.0, 214.9) | 210.3 (168.1, 263.2) | 52.8 (41.8, 66.7) | 31.0 (24.5, 39.2) | 210.1 (171.4, 257.7) |
| HAI titer ≥1:40,^c^ % (95% CI)^d^ | 92.5 (87.7, 95.8) | 97.8 (94.3, 99.4) | 76.1 (69.3, 82.0) | 56.7 (49.3, 63.9) | 97.9 (94.7, 99.4) | 94.3 (87.1, 98.1) | 96.7 (90.6, 99.3) | 68.2 (57.2, 77.9) | 48.3 (37.4, 59.2) | 97.8 (92.1, 99.7) |
| GMFR (95% CI)^b^ | 31.4 (26.8, 36.8) | 43.8 (38.3, 50.0) | 11.2 (9.8, 12.9) | 7.0 (6.0, 8.2) | 48.4 (41.8, 56.1) | 31.3 (25.3, 38.5) | 38.1 (30.3, 47.8) | 10.5 (8.3, 13.2) | 6.2 (4.9, 7.9) | 37.3 (30.0, 46.2) |
| Seroconversion,^e^ % (95% CI)^d^ | 92.5 (87.7, 95.8) | 97.8 (94.3, 99.4) | 76.1 (69.3, 82.0) | 56.7 (49.3, 63.9) | 97.4 (93.9, 99.1) | 94.3 (87.1, 98.1) | 96.7 (90.6, 99.3) | 68.2 (57.2, 77.9) | 48.3 (37.4, 59.2) | 96.6 (90.5, 99.3) |
| Day 205, n | 186 | 182 | 181 | 186 | 180 | 86 | 89 | 85 | 85 | 89 |
| GMT (95% CI)^b^ | 41.3 (34.9, 48.9) | 59.9 (51.3, 69.9) | 14.1 (12.3, 16.1) | 10.5 (9.3, 11.9) | 54.7 (46.5, 64.5) | 52.6 (41.0, 67.6) | 65.8 (51.7, 83.9) | 13.7 (11.1, 16.9) | 9.4 (7.9, 11.2) | 50.5 (39.6, 64.4) |
| HAI titer ≥1:40,^c^ % (95% CI)^d^ | 61.8 (54.4, 68.8) | 75.8 (68.9, 81.9) | 20.4 (14.8, 27.1) | 12.9 (8.4, 18.6) | 73.3 (66.2, 79.6) | 72.1 (61.4, 81.2) | 78.7 (68.7, 86.6) | 23.5 (15.0, 34.0) | 10.6 (5.0, 19.2) | 65.2 (54.3, 75.0) |
| GMFR (95% CI)^b^ | 8.0 (6.8, 9.4) | 11.6 (9.9, 13.5) | 2.8 (2.4, 3.2) | 2.1 (1.9, 2.4) | 9.6 (8.1, 11.4) | 9.4 (7.4, 12.0) | 11.9 (9.4, 15.1) | 2.7 (2.2, 3.4) | 1.9 (1.6, 2.2) | 9.0 (7.0, 11.5) |
| Seroconversion,^e^ % (95% CI)^d^ | 61.8 (54.4, 68.8) | 75.8 (68.9, 81.9) | 20.4 (14.8, 27.1) | 12.9 (8.4, 18.6) | 71.7 (64.5, 78.1) | 72.1 (61.4, 96.1) | 78.7 (68.7, 86.6) | 23.5 (15.0, 34.0) | 10.6 (5.0, 19.2) | 64.0 (53.2, 73.9) |
| **Neuraminidase inhibition assay^a^** | | | | | | | | | | |
| Day 1 (baseline), n | 191 | - | 190 | - | - | 88 | - | 87 | - | - |
| GMT (95% CI)^b^ | 5.5 (5.2, 5.9) | - | 5.1 (5.0, 5.2) | - | - | 8.7 (6.8, 11.1) | - | 6.3 (5.5, 7.2) | - | - |
| Day 8, n | 190 | - | 189 | - | - | 87 | - | 85 | - | - |
| GMT (95% CI)^b^ | 6.2 (5.6, 6.8) | - | 5.5 (5.1, 5.9) | - | - | 9.2 (7.1, 11.8) | - | 6.7 (5.7, 7.8) | - | - |
| GMFR (95% CI)^b^ | 1.1 (1.1, 1.2) | - | 1.1 (1.0, 1.2) | - | - | 1.1 (1.0, 1.1) | - | 1.1 (1.0, 1.1) | - | - |
| ≥4-fold rise from baseline, % (95% CI)^f^ | 3.2 (1.2, 6.7) | - | 1.6 (0.3, 4.6) | - | - | 1.1 (0, 6.2) | - | 0 (0, 4.2) | - | - |
| Day 22, n | 191 | - | 190 | - | - | 88 | - | 87 | - | - |
| GMT (95% CI)^b^ | 30.7 (26.2, 35.9) | - | 17.0 (14.6, 19.7) | - | - | 31.8 (24.1, 42.0) | - | 14.1 (11.1, 17.8) | - | - |
| GMFR (95% CI)^b^ | 5.6 (4.8, 6.5) | - | 3.4 (2.9, 3.9) | - | - | 3.7 (2.9, 4.6) | - | 2.2 (1.9, 2.7) | - | - |
| ≥4-fold rise from baseline, % (95% CI)^f^ | 52.9 (45.5, 60.1) | - | 29.5 (23.1, 36.5) | - | - | 38.6 (28.4, 49.6) | - | 13.8 (7.3, 22.9) | - | - |
| Day 29, n | 184 | - | 188 | - | - | 87 | - | 82 | - | - |
| GMT (95% CI)^b^ | 217.9 (187.2, 253.7) | - | 361.4 (303.2, 430.7) | - | - | 167.8 (133.1, 211.6) | - | 230.1 (171.0, 309.8) | - | - |
| GMFR (95% CI)^b^ | 39.2 (33.5, 45.9) | - | 71.2 (59.8, 84.8) | - | - | 19.2 (14.6, 25.2) | - | 37.0 (27.2, 50.2) | - | - |
| ≥4-fold rise from baseline, % (95% CI)^f^ | 96.2 (92.3, 98.5) | - | 98.4 (95.4, 99.7) | - | - | 86.2 (77.1, 92.7) | - | 89.0 (80.2, 94.9) | - | - |
| Day 43, n | 186 | - | 188 | - | - | 87 | - | 85 | - | - |
| GMT (95% CI)^b^ | 150.7 (132.4, 171.6) | - | 197.4 (168.8, 230.8) | - | - | 139.7 (115.1, 169.6) | - | 152.4 (118.1, 196.5) | - | - |
| GMFR (95% CI)^b^ | 27.2 (23.7, 31.1) | - | 38.9 (33.3, 45.4) | - | - | 16.0 (12.5, 20.5) | - | 24.1 (18.5, 31.3) | - | - |
| ≥4-fold rise from baseline, % (95% CI)^f^ | 95.7 (91.7, 98.1) | - | 95.2 (91.1, 97.8) | - | - | 86.2 (77.1, 92.7) | - | 91.8 (83.8, 96.6) | - | - |
| Day 205, n | 186 | - | 180 | - | - | 86 | - | 85 | - | - |
| GMT (95% CI)^b^ | 96.7 (83.3, 112.3) | - | 185.9 (160.8, 215.0) | - | - | 110.4 (86.1, 141.7) | - | 165.3 (131.7, 207.5) | - | - |
| GMFR (95% CI)^b^ | 17.4 (15.0, 20.3) | - | 36.6 (31.7, 42.3) | - | - | 12.6 (9.6, 16.5) | - | 26.1 (20.6, 33.0) | - | - |
| ≥4-fold rise from baseline, % (95% CI)^f^ | 87.6 (82.0, 92.0) | - | 95.6 (91.4, 98.1) | - | - | 81.4 (71.6, 89.0) | - | 92.9 (85.3, 97.4) | - | - |
| **Neutralization antibody^a^ by microneutralization assay** | | | | | | | | | | |
| Day 1 (baseline), n | 191 | 189 | 190 | 196 | 194 | 88 | 90 | 87 | 88 | 89 |
| GMT (95% CI)^b^ | 5.0 (5.0, 5.0) | 5.0 (5.0, 5.1) | 8.1 (7.8, 8.4) | 8.0 (7.8, 8.2) | 6.5 (6.2, 6.8) | 5.5 (5.1, 5.8) | 5.9 (5.2, 6.8) | 7.9 (7.7, 8.1) | 8.4 (7.9, 8.9) | 6.2 (5.9, 6.4) |
| Day 8, n | 190 | 189 | 189 | 194 | 193 | 87 | 90 | 86 | 88 | 89 |
| GMT (95% CI)^b^ | 7.5 (6.8, 8.3) | 7.3 (6.5, 8.2) | 10.5 (9.3, 12.0) | 9.3 (8.4, 10.4) | 9.1 (8.0, 10.4) | 9.4 (7.9, 11.2) | 9.4 (7.9, 11.3) | 8.4 (8.0, 8.9) | 9.7 (8.7, 10.8) | 8.7 (7.3, 10.3) |
| GMFR (95% CI)^b^ | 1.51 (1.36, 1.66) | 1.46 (1.30, 1.64) | 1.3 (1.16, 1.46) | 1.18 (1.07, 1.29) | 1.4 (1.3, 1.6) | 1.72 (1.45, 2.04) | 1.59 (1.29, 1.97) | 1.06 (1.01, 1.12) | 1.16 (1.07, 1.26) | 1.4 (1.2, 1.7) |
| ≥4-fold rise from baseline, % (95% CI)^d^ | 3.7 (1.5, 7.4) | 4.2 (1.8, 8.2) | 3.7 (1.5, 7.5) | 3.1 (1.1, 6.6) | 7.3 (4.0, 11.9) | 5.7 (1.9, 12.9) | 7.8 (3.2, 15.4) | 0 (0.0, 4.2) | 0 (0.0, 4.1) | 6.7 (2.5, 14.1) |
| Day 22, n | 191 | 189 | 190 | 196 | 194 | 88 | 90 | 87 | 88 | 89 |
| GMT (95% CI)^b^ | 41.4 (36.3, 47.2) | 40.3 (35.0, 46.3) | 38.0 (32.1, 44.9) | 32.9 (28.3, 38.3) | 19.0 (16.5, 21.9) | 41.2 (33.5, 50.6) | 37.7 (30.8, 46.2) | 21.4 (17.6, 26.1) | 19.2 (15.9, 23.1) | 15.2 (12.3, 18.7) |
| GMFR (95% CI)^b^ | 8.27 (7.25, 9.44) | 8.02 (6.97, 9.22) | 4.69 (4.00, 5.51) | 4.13 (3.56, 4.79) | 2.9 (2.6, 3.4) | 7.56 (6.17, 9.25) | 6.36 (5.01, 8.06) | 2.70 (2.23, 3.27) | 2.29 (1.91, 2.75) | 2.5 (2.0, 3.1) |
| ≥4-fold rise from baseline, % (95% CI)^d^ | 50.3 (43.0, 57.6) | 47.6 (40.3, 55.0) | 24.7 (18.8, 31.5) | 27.6 (21.4, 34.4) | 16.5 (11.6, 22.5) | 44.3 (33.7, 55.3) | 41.1 (30.8, 52.0) | 11.5 (5.7, 20.1) | 9.1 (4.0, 17.1) | 14.6 (8.0, 23.7) |
| Day 29, n | 185 | 183 | 188 | 188 | 189 | 87 | 88 | 82 | 88 | 87 |
| GMT (95% CI)^b^ | 147.9 (129.3, 169.2) | 244.4 (210.3, 284.1) | 211.2 (177.8, 250.8) | 235.4 (196.3, 282.4) | 77.3 (66.3, 90.1) | 136.4 (109.8, 169.3) | 237.2 (191.9, 293.0) | 122.8 (94.7, 159.4) | 162.0 (128.9, 203.6) | 52.7 (42.7, 65.1) |
| GMFR (95% CI)^b^ | 29.58 (25.86, 33.85) | 48.67 (41.88, 56.56) | 26.08 (22.09, 30.80) | 29.48 (24.59, 35.34) | 11.9 (10.2, 14.0) | 24.98 (20.25, 30.83) | 39.84 (31.60, 50.21) | 15.60 (12.02, 20.23) | 19.34 (15.25, 24.54) | 8.6 (6.9, 10.6) |
| ≥4-fold rise from baseline, % (95% CI)^d^ | 90.8 (85.7, 94.6) | 95.1 (90.9, 97.7) | 84.0 (78.0, 89.0) | 85.6 (79.8, 90.3) | 67.2 (60.0, 73.8) | 88.5 (79.9, 94.3) | 92.0 (84.3, 96.7) | 70.7 (59.6, 80.3) | 80.7 (70.9, 88.3) | 57.5 (46.4, 68.0) |
| Day 43, n | 186 | 178 | 188 | 187 | 189 | 87 | 90 | 85 | 87 | 89 |
| GMT (95% CI)^b^ | 124.4 (108.8, 142.2) | 213.7 (187.7, 243.2) | 167.3 (144.1, 194.2) | 209.9 (180.7, 243.9) | 76.0 (66.0, 87.6) | 129.6 (106.9, 157.2) | 239.9 (194.7, 295.6) | 110.0 (88.5, 136.6) | 158.1 (123.6, 202.1) | 60.9 (51.2, 72.5) |
| GMFR (95% CI)^b^ | 24.88 (21.77, 28.44) | 42.55 (37.37, 48.44) | 20.66 (17.91, 23.82) | 26.28 (22.63, 30.52) | 11.7 (10.1, 13.6) | 23.75 (19.62, 28.75) | 40.45 (32.14, 50.92) | 13.85 (11.16, 17.18) | 18.86 (14.77, 24.07) | 9.9 (8.3, 11.8) |
| ≥4-fold rise from baseline, % (95% CI)^d^ | 90.9 (85.8, 94.6) | 97.2 (93.6, 99.1) | 80.3 (73.9, 85.7) | 88.2 (82.7, 92.5) | 69.3 (62.2, 75.8) | 90.8 (82.7, 95.9) | 93.3 (86.1, 97.5) | 67.1 (56.0, 76.9) | 81.6 (71.9, 89.1) | 64.0 (53.2, 73.9) |
| Day 205, n | 186 | 182 | 181 | 186 | 180 | 86 | 89 | 85 | 85 | 89 |
| GMT (95% CI)^b^ | 29.0 (25.7, 32.8) | 44.0 (38.8, 49.7) | 47.4 (40.7, 55.1) | 50.1 (42.9, 58.4) | 21.5 (18.9, 24.5) | 36.8 (30.3, 44.7) | 62.1 (50.5, 76.4) | 34.1 (28.5, 41.0) | 42.6 (34.4, 52.7) | 21.4 (17.7, 26.0) |
| GMFR (95% CI)^b^ | 5.80 (5.14, 6.56) | 8.75 (7.74, 9.90) | 5.84 (5.06, 6.75) | 6.30 (5.40, 7.34) | 3.3 (2.9, 3.8) | 6.73 (5.50, 8.24) | 10.45 (8.36, 13.07) | 4.30 (3.59, 5.15) | 5.07 (4.08, 6.30) | 3.5 (2.9, 4.2) |
| ≥4-fold rise from baseline, % (95% CI)^d^ | 31.7 (25.1, 38.9) | 56.6 (49.1, 63.9) | 37.0 (30.0, 44.5) | 40.3 (33.2, 47.7) | 15.6 (10.6, 21.7) | 44.2 (33.5, 55.3) | 62.9 (52.0, 72.9) | 28.2 (19.0, 39.0) | 38.8 (28.4, 50.0) | 18.0 (10.6, 27.5) |

Abbreviations: GMFR, geometric mean fold rise; GMT, geometric mean titer; LLOQ, lower limit of quantification; NA, not available; ULOQ, upper limit of quantification.

^a^H5N8 and H5-only: A/Astrakhan/3212/2020; H7N9 and H7-only: A/Anhui/1/2013; H5-only-GC: A/chicken/Ghana/2021.

^b^95% CI was calculated based on the *t* distribution of the log-transformed values or the difference in the log-transformed values for the GMT and GMFR, respectively, then back-transformed to the original scale for presentation.

^c^HAI titers ≥1:40 are associated with seroprotection.

^d^95% CI calculated using the Clopper-Pearson methodology.

^e^Percentage of participants with seroconversion was defined as titer ≥1:40 if baseline was <1:10 or a 4-fold or greater rise if baseline was ≥1:10 in HAI antibodies measured by the HAI assay.

^f^≥4-fold rise from baseline at a participant level is defined as a ≥4 × LLOQ for participants with baseline antibody levels < LLOQ, or a 4 × or higher antibody level ratio in participants with a baseline antibody level ≥LLOQ.

**References**

1.	World Health Organization. Summary of status of development and availability of A(H5) non–A(H5N1) candidate vaccine viruses and potency testing reagents. Available at: <https://cdn.who.int/media/docs/default-source/cvv-southern-hemisphere-2025/h5-non-h5n1_cvv_-20240927.pdf?sfvrsn=11d78877_1>. Accessed 17 October.

2.	World Health Organization. Summary of status of development and availability of avian influenza A(H7N9) candidate vaccine viruses and potency testing reagents. Available at: <https://cdn.who.int/media/docs/default-source/cvv-southern-hemisphere-2025/h7n9_summary_a_h7n9_cvv_20240927.pdf?sfvrsn=18c11b44_1>. Accessed 17 October.

3.	World Health Organization. Summary of status of development and availability of A(H5N1) candidate vaccine viruses and potency testing reagents. Available at: <https://cdn.who.int/media/docs/default-source/cvv-southern-hemisphere-2025/h5n1_summary_a_h5n1_cvv_20240927.pdf?sfvrsn=f7f9ff20_1>. Accessed 17 October.

4.	Uyeki Timothy M, Milton S, Abdul Hamid C, et al. Highly pathogenic avian influenza A(H5N1) virus infection in a dairy farm worker. N Engl J Med **2024**; 390(21): 2028-9.

5.	Center for Disease Control and Prevention. Technical Report: June 2024 Highly Pathogenic Avian Influenza A(H5N1) Viruses. Available at: <https://www.cdc.gov/bird-flu/php/technical-report/h5n1-06052024.html>. Accessed 18 March.

6.	World Health Organization. Serological detection of avian influenza A(H7N9) virus infections by modified horse red blood cells haemagglutination-inhibition assay. Available at: <https://cdn.who.int/media/docs/default-source/influenza/avian-and-other-zoonotic-influenza/h7n9-technical-guidance/cnic_serological_diagnosis_hai_a_h7n9_20131220.pdf?sfvrsn=ae4773a5_10> Accessed 18 March.

7.	Prevato M, Ferlenghi I, Bonci A, et al. Expression and characterization of recombinant, tetrameric and enzymatically active influenza neuraminidase for the setup of an enzyme-linked lectin-based assay. PLoS One **2015**; 10(8): e0135474.

8.	Bernard MC, Waldock J, Commandeur S, et al. Validation of a harmonized enzyme-linked-lectin-assay (ELLA-NI) based neuraminidase inhibition assay standard operating procedure (SOP) for quantification of N1 influenza antibodies and the use of a calibrator to improve the reproducibility of the ELLA-NI with reverse genetics viral and recombinant neuraminidase antigens: A FLUCOP collaborative study. Front Immunol **2022**; 13: 909297.

9.	Gao J, Couzens L, Eichelberger MC. Measuring influenza neuraminidase inhibition antibody titers by enzyme-linked lectin assay. J Vis Exp **2016**; (115): 54573.

10.	World Health Organization. Serological diagnosis of influenza by microneutralization assay. Available at: Microsoft Word - Serological diagnosis for web 6_12_2010.doc Accessed 18 March.

11.	U.S. Food and Drug Administration. Toxicity grading scale for healthy adult and adolescent volunteers enrolled in preventive vaccine clinical trials. Available at: <https://www.fda.gov/regulatory-information/search-fda-guidance-documents/toxicity-grading-scale-healthy-adult-and-adolescent-volunteers-enrolled-preventive-vaccine-clinical>. Accessed 18 March.
